# SeSaMe: Metagenome Sequence Classification of Arbuscular Mycorrhizal Fungi-associated Microorganisms

**DOI:** 10.1016/j.gpb.2018.07.010

**Published:** 2020-12-18

**Authors:** Jee Eun Kang, Antonio Ciampi, Mohamed Hijri

**Affiliations:** 1 Institut de Recherche en Biologie Végétale, Département de Sciences Biologiques, Université de Montréal, Montréal, QC H1X 2B2, Canada; 2 Department of Epidemiology, Biostatistics and Occupational Health, McGill University, Montréal, QC H3A 1A2, Canada

**Keywords:** SeSaMe, Spore-associated symbiotic microbes, Arbuscular mycorrhizal fungi, Taxonomic classification, 3-codon DNA 9-mer

## Abstract

**Arbuscular mycorrhizal fungi** (AMF) are plant root symbionts that play key roles in plant growth and soil fertility. They are obligate biotrophic fungi that form coenocytic multinucleated hyphae and spores. Numerous studies have shown that diverse microorganisms live on the surface of and inside their mycelia, resulting in a metagenome when whole-genome sequencing (WGS) data are obtained from sequencing AMF cultivated *in vivo.* The metagenome contains not only the AMF sequences, but also those from associated microorganisms. In this study, we introduce a novel bioinformatics program, **Spore-associated Symbiotic Microbes** (**SeSaMe**), designed for **taxonomic classification** of short sequences obtained by next-generation DNA sequencing. A genus-specific usage bias database was created based on amino acid usage and codon usage of a three consecutive codon DNA 9-mer encoding an amino acid trimer in a protein secondary structure. The program distinguishes between coding sequence (CDS) and non-CDS, and classifies a query sequence into a genus group out of 54 genera used as reference. The mean percentages of correct predictions of the CDS and the non-CDS test sets at the genus level were 71% and 50% for bacteria, 68% and 73% for fungi (excluding AMF), and 49% and 72% for AMF (*Rhizophagus irregularis*), respectively. SeSaMe provides not only a means for estimating taxonomic diversity and abundance but also the gene reservoir of the reference taxonomic groups associated with AMF. Therefore, it enables users to study the symbiotic roles of associated microorganisms. It can also be applicable to other microorganisms as well as soil metagenomes. SeSaMe is freely available at www.fungalsesame.org.

## Introduction

Arbuscular mycorrhizal fungi (AMF) are plant root inhabiting fungi, of the subphylum Glomeromycotina, which form symbioses with more than 80% of vascular plants worldwide [1]. They supply plants with essential nutrients particularly phosphorus and nitrogen, protect them against soil-borne pathogens, and alleviate their abiotic stresses [1], [2], [3]. Therefore, AMF-based inoculants have been applied in agriculture as a biofertilizer and in phytoremediation for cleaning up contaminated soil [2], [4], [5], [6], [7]. Despite the ecological, agricultural, and environmental importance of AMF, their genetics is poorly understood due to their complex genome organization. They form coenocytic hyphae, reproduce through multinucleated asexual spores, and are strict symbionts [8]. Furthermore, it is suggested that AMF are heterokaryons, although this is under debate [9]. In addition, numerous studies reported that bacteria and fungi inhabit the surface and the interior of mycelia and spores [10], [11], [12], [13], [14]. In 2012 and 2013, Tisserant et al. [15], [16] separately published the transcriptome and genome of the AMF *Rhizophagus irregularis* cultivated *in vitro*. However, only a few AMF taxa are able to grow in axenic *in vitro* systems with transformed roots as a host. Thus, whole-genome sequencing (WGS) data from AMF spore DNA originating from *in vivo* cultures (a conventional cultivation method in a pot culture with a host plant), contain a substantial number of non-AMF DNA sequences, but do provide important information on the microbial communities associated with AMF. In contrast, WGS data from *in vitro* petri-dishes contain fewer non-AMF sequences, because antibiotics are used to initiate axenic cultures [17].

Taxonomic classification of WGS data obtained from AMF cultivated *in vivo* using current bioinformatics approaches is challenging because these data represent a complex metagenome containing sequences of prokaryotic and eukaryotic microorganisms. Two major approaches for taxonomic classification of random whole metagenome sequencing data (*e.g.*, whole metagenome shotgun sequencing data) include composition-based methods and similarity-based search methods [18], [19]. The latter ones include BLAST and its sister programs that are adequate for inferring functions of a query sequence [19], [20]. Nevertheless, they have limitations in taxonomic classification, because they calculate scores based on a 20 by 20 matrix containing the overall rates of the 20 amino acid substitutions created from the most conserved regions of proteins. The same matrix is applied to all types of query sequences, irrespective of functions, structures, and taxonomic groups. However, due to a lack of bioinformatics tools for analyzing random whole metagenome sequencing data, similarity-based search methods have been commonly used for taxonomic classification. In addition to similarity-based search methods, taxonomic classification pipelines for analyzing targeted metagenome sequencing data (*e.g.*, 16S rRNA gene-based metagenome sequencing data) have been widely used for analyzing random whole metagenome sequencing data in combination with homology search program. Numerous repository databases and pipelines have been developed based on the 16S rRNA gene. However, a previous study has reported the horizontal gene transfer of 16S rRNA genes in prokaryotic organisms and the multiple heterogeneous rRNA genes within a single prokaryotic cell [21]. Therefore, they may cause misrepresentation of data if they are not properly dealt with, which may result in erroneous taxonomic classification.

Composition-based methods utilize unique sequence properties such as codon usage bias, compositional patterns in nucleotide sequences (k-mers), and GC content that have been widely used for studying microbial genome evolution in areas of bioinformatics [18], [22], [23], [24], [25], [26]. K-mers are subsequences of length k in a DNA sequence (*e.g.*, tetramer or 4-mer: ATGT). Composition-based methods using k-mers have been employed in bioinformatics programs for taxonomic classification of random whole metagenome sequencing data [27]. They have a number of advantages over similarity-based search methods. It is estimated that more than 99% of existing microorganisms cannot be cultured in laboratory conditions [28], and microbial sequences available in bioinformatics databases represent only a tiny fraction of the diversity of existing microorganisms. Therefore, composition-based methods, which do not require sequence alignments but make predictions based on microorganism's unique sequence signatures, supposedly excel in taxonomical classification of novel sequences. However, existing bioinformatics programs based on composition-based methods are designed for prokaryotic organisms and their utilization in fungi is inefficient.

**Figure 1:**
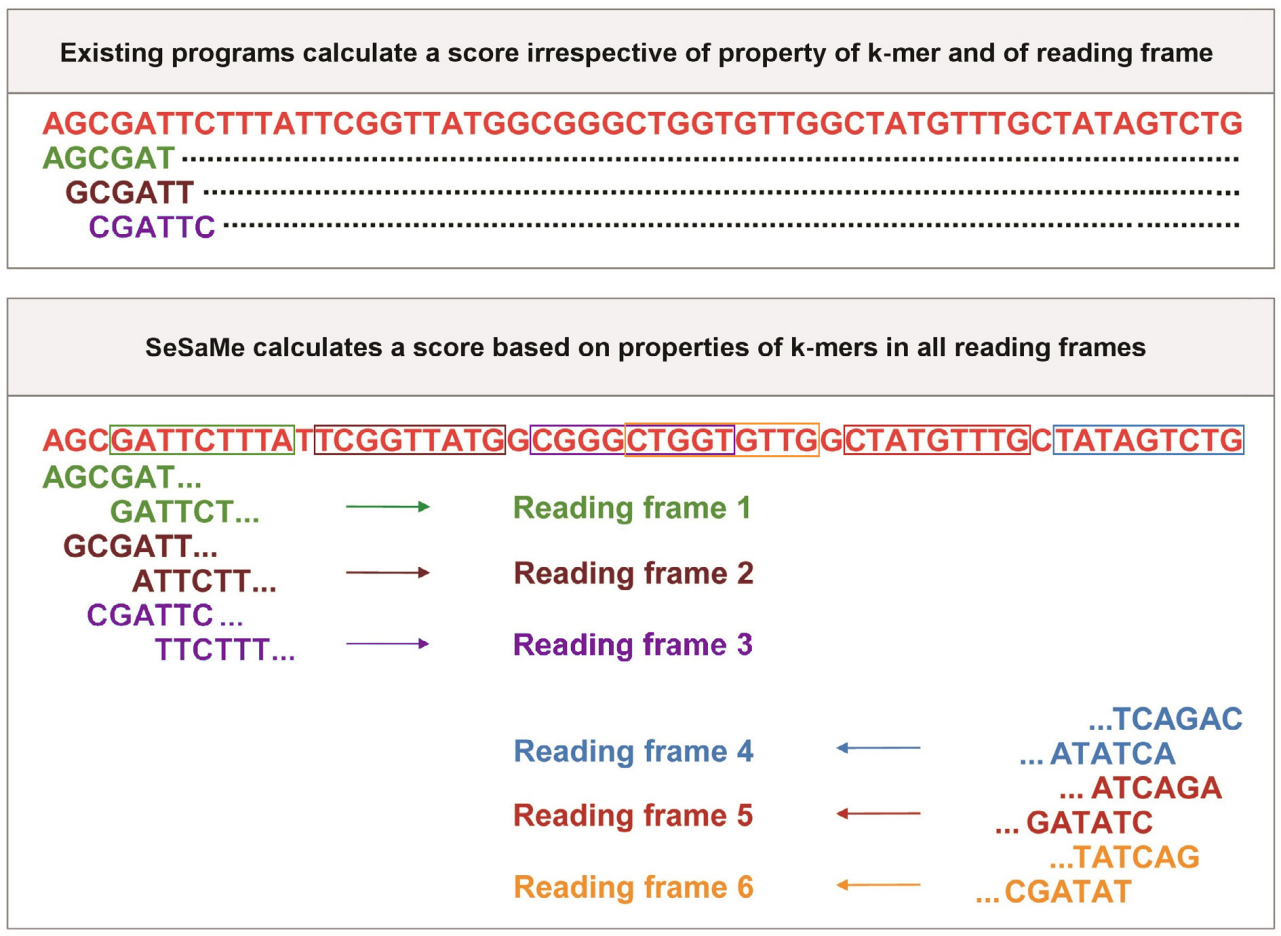
**Unique advantage of SeSaMe over existing programs** Existing programs calculate a score based on the frequencies of k-mers identified in a query sequence irrespective of properties of the k-mers, or their reading frames. In contrast, SeSaMe identifies k-mers that encode the amino acids of protein secondary structures in each reading frame. In the figure, matching 3-codon DNA 9-mers of the Trimer Ref. DB are marked with rectangles, where a color of the rectangle indicates a reading frame. SeSaMe calculates scores based on the 3-codon usages and the A.A. Trimer usages of the matching 3-codon DNA 9-mers in each reading frame. It classifies a query sequence into a taxonomic group based on the six scores computed from all reading frames. All sequences in this figure are randomly generated for illustration purposes only. SeSaMe, Spore-associated Symbiotic Microbes; A.A., amino acid; Trimer Ref. DB, trimer reference sequence database.

In this study, we introduce a novel bioinformatics program for random whole metagenome sequence classification, SeSaMe (Spore-associated Symbiotic Microbes). It provides a means for estimating taxonomic diversity and abundance, as well as, the reservoir of genes of reference taxonomic groups in AMF metagenome. It therefore enables users to study symbiotic roles of taxonomic groups associated with AMF. In order to filter complex evolutionary signals and obtain comparable evolutionary footprints, we calculated codon usage bias based on the amino acid usage and the codon usage of a 3-codon DNA 9-mer that encodes three consecutive amino acids located in a protein secondary structure. We joined three consecutive codons into one unit, and calculated the unit’s relative frequency among synonymous 3-codon DNA 9-mers, which will be hereafter referred to as 3-codon usage. 3-codon usage has higher resolution than mono codon usage in assessing the differences among taxonomic groups because evolutionary forces acting on a codon and its encoded amino acid vary widely across protein secondary structures as well as across taxonomic groups. For example, the evolutionary forces acting on the codon AAA, encoding the amino acid Lysine (K) in TGG***AAA***GTG (WKV), have been different from the evolutionary forces acting on the codon AAA in GAC***AAA***GAA (DKE). We found that 3-codon usage of a 3-codon DNA 9-mer belonging to a protein secondary structure is a taxonomically unique sequence property. SeSaMe calculates a score based on six sets of 3-codon DNA 9-mers from all reading frames (Figure 1), and distinguishes between coding sequence (CDS) and non-CDS. It has an advantage over existing composition-based methods that do not identify nucleotide subsequences with structural roles, or do not consider the biological importance of codons and reading frames. SeSaMe is freely available at www.fungalsesame.org.

## Method

### Bacterial and fungal sequence databases

We selected bacterial genera that were dominant in soil based on a literature review [10], [28], [29], [30], [31], [32]. While NCBI offered a broad selection of more than 2300 completely sequenced bacterial genomes, we did not have many choices for the majority of fungal phyla. Most of the completely sequenced fungal genomes in NCBI or JGI were Dikarya, while we needed diverse fungal genomes covering Mucoromycotina, AMF, Blastocladiomycota, Neocallimastigomycota, Microsporidia, and Chytridiomycota. We assigned the completely sequenced genomes of 444 bacteria and 11 fungi, including *R. irregularis*, to 45 bacterial and 9 fungal genera, respectively, and created CDS and non-CDS databases per genus based on CDS lists provided by NCBI, JGI, and Tisserant et al. [16]. The number of genomes per genus varied from 1 to 81, depending on their availability in public databases. The total number of the bacterial genes per genus and the total number of the fungal genes and introns per genus are shown in Tables S1 and S2, respectively. Sequences with an ambiguous nucleotide or with a length shorter than nine — the minimum length of nucleotides required for a 3-codon DNA 9-mer — were excluded. *Cryptococcus* and Agaricomycetes (*Phanerochaete*, *Scleroderma*, and *Sebacina*) belong to the same subdivision, Agaricomycotina, and were grouped together in order to simplify the analysis.

### Database design

**Figure 2:**
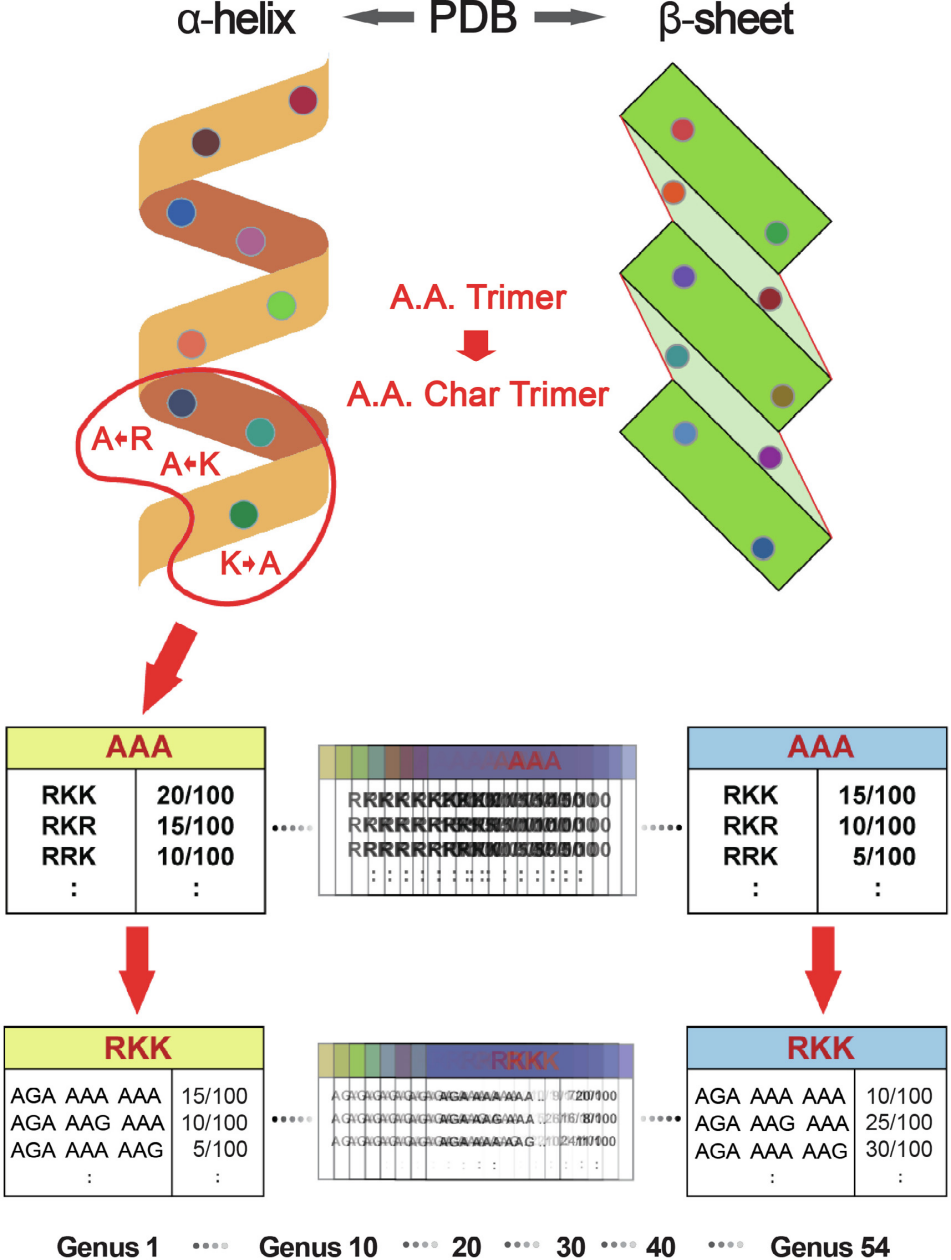
**Database design** In this figure, A.A. Trimer Usage Table consists of the A.A. Trimer usages of the multiple members, with RKK, RKR, and RRK belonging to the same A.A. Char Trimer, AAA. 3-codon Usage Table consists of the 3-codon usages of the synonymous 3-codon DNA 9-mers encoding the A.A. Trimer, RKK (*e.g.*, AGA AAA AAA). The trimer usage bias of AGA AAA AAA is the multiplication of the A.A. Trimer usage of RKK and the 3-codon usage of AGA AAA AAA. All sequences and usage information in this figure are not real, but randomly chosen for illustration purposes only. Char, characteristic.

For selecting a parameter k of k-mer, we chose 3-codon DNA 9-mer as the length of amino acids and of nucleotides, considering the approximate number of amino acids required to form a helical turn in helix and a beta-strand. The program consists of two main components: databases and scoring methods. The major distinguishing feature is the trimer reference sequence database (Trimer Ref. DB). 126,093 Protein Data Bank (PDB) entry files were processed with in-house developed parsing programs to extract 7674 amino acid trimers (A.A. Trimers), subunits of protein secondary structures, which were assigned to the sequence variable — A.A. Trimer [33]. 224,383 3-codon DNA 9-mers, encoding 7674 A.A. Trimers, were assigned to the sequence variable — 3-codon DNA 9-mer. In Trimer Ref. DB, the sequence variables, A.A. Char Trimer (amino acid characteristic trimer), A.A. Trimer, and 3-codon DNA 9-mer, form a three-level hierarchy where A.A. Char Trimer is the highest level (Figure 2). To create A.A. Char, first, we assigned amino acids with similar properties into one group according to polarity and charge of their side chain, and secondly subdivided each group according to their volume (Table 1). Cysteine, glycine, histidine, methionine, and proline have special properties. Therefore, each of them was assigned as a sole member of an A.A. Char group. Generally, multiple A.A. Trimers with similar properties belong to one A.A. Char Trimer. An A.A. Char Trimer and an A.A. Trimer have an A.A. Trimer table and a 3-codon DNA 9-mer table containing multiple members, respectively (Figure 2).

**Table 1:** **Conversion table from A.A. to A.A. Char**

**A.A. Char**	**A.A**.	**Property**	**A.A. Char**	**A.A.**	**Property**
A	K, R	Positively charged	G	G	Special
B	H	Special	H	P	Special
C	D, E	Negatively charged	I	M	Special
D	S, T	Polar uncharged; smaller volume	J	A, I, L, V	Hydrophobic; smaller volume
E	N, Q	Polar uncharged; larger volume	K	F, W, Y	Hydrophobic; larger volume
F	C	Special	L	*	Stop codon

*Note*: A.A. residues were grouped according to pKa values of their side chains, charges at physiological pH (7.4), and volumes. A.A., amino acid; Char, characteristic.

Genus-specific usage bias database (Genus-specific DB) contains the main numerical variable — trimer usage bias. Trimer usage bias is calculated by multiplying the A.A. Trimer usage of A.A. Trimer by the 3-codon usage of 3-codon DNA 9-mer in Trimer Ref. DB (Figure 2). There are 54 CDS Genus-specific DBs and the same number of non-CDS Genus-specific DBs in the program. Each CDS Genus-specific DB contains 1296 A.A. Trimer Usage Tables and 7674 3-codon Usage Tables created based on the CDS database. Non-CDS Genus-specific DB contains the same number of tables derived from the same sequence variables of the Trimer Ref. DB for cost-effective CDS and non-CDS classification. Because SeSaMe compares frequency information of 54 genera calculated based on the same standard genetic code table for the same 3-codon DNA 9-mers, inaccuracy in calculating trimer usage bias of non-CDS is assumed to be insignificant.

### Scoring methods

We developed two scoring methods, and each equipped with a *P* value scoring method. The trimer usage probability scoring method classifies a query sequence into one out of 54 genus references, while the rank probability scoring method classifies a query sequence into one out of 13 taxonomic groups: Clostridia, Bacilli, Oscillatoriophycideae, Nostocales, Acidobacteriales, Betaproteobacteria, Deltaproteobacteria, Gammaproteobacteria, Alphaproteobacteria, Actinobacteria, AMF (*R. irregularis*), Agaricomycotina, and Pezizomycotina. To avoid word repetition, these taxonomic groups will be hereafter referred to as 13 taxon groups, and in the same order as shown in the list above.

#### Trimer usage probability scoring method

**Figure 3:**
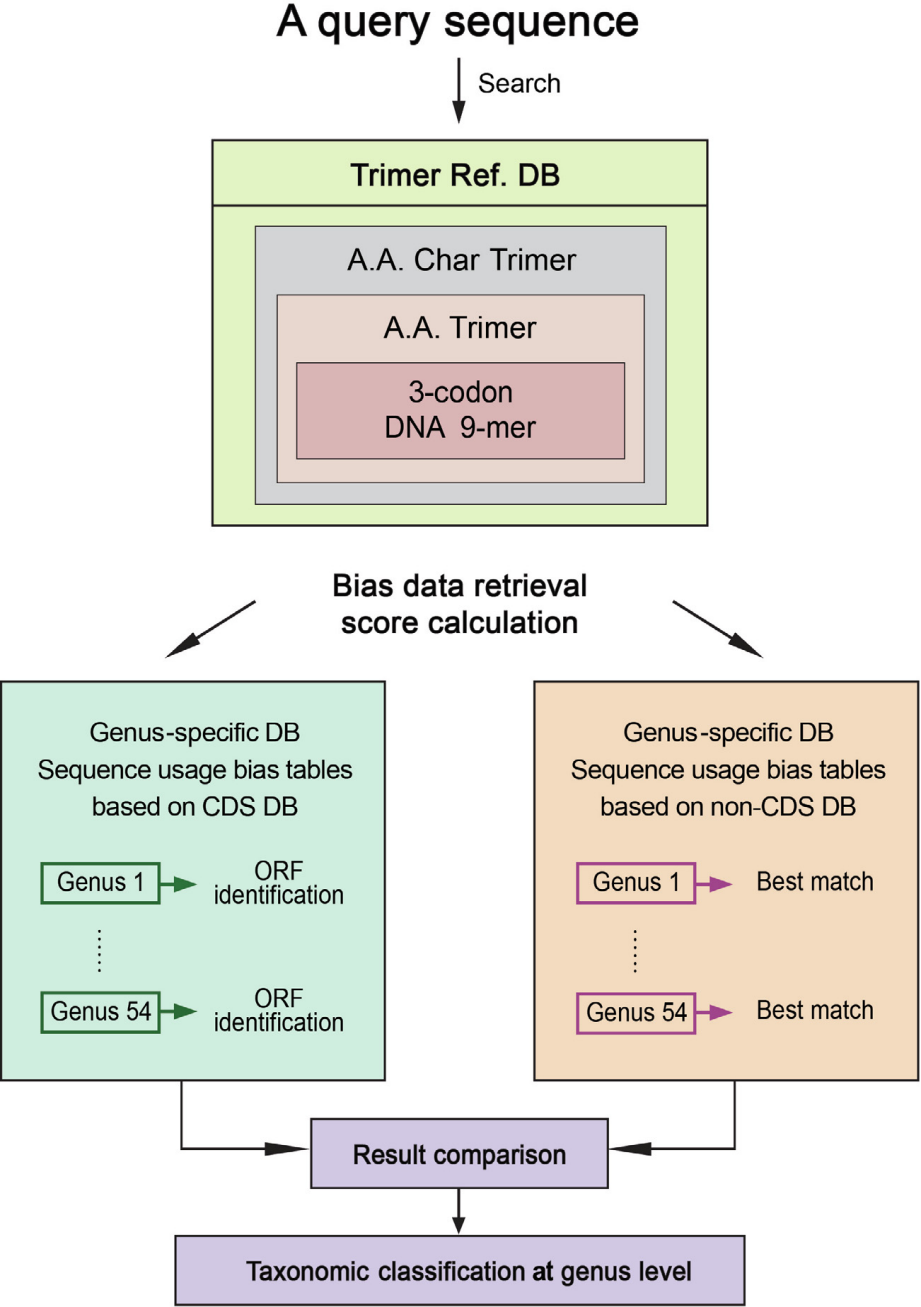
**Flow chart of the program**

This method converts 3-codon DNA 9-mers in a query sequence into A.A. Char Trimers and identifies those with structural roles by searching them against Trimer Ref. DB. For each matching A.A. Char Trimer, the method first searches a matching A.A. Trimer, and second, a matching 3-codon DNA 9-mer in Trimer Ref. DB (Figure 3). It retrieves trimer usage biases of the matching 3-codon DNA 9-mers from CDS Genus-specific DB per reading frame of a query sequence. It repeats the same process with non-CDS Genus-specific DB. It then compares scores from CDS and non-CDS Genus-specific DBs, and selects a genus with the highest score (Figure 3). Users are provided with an option to include genera whose scores have little difference from the highest score.

#### Rank probability scoring method

**Figure 4:**
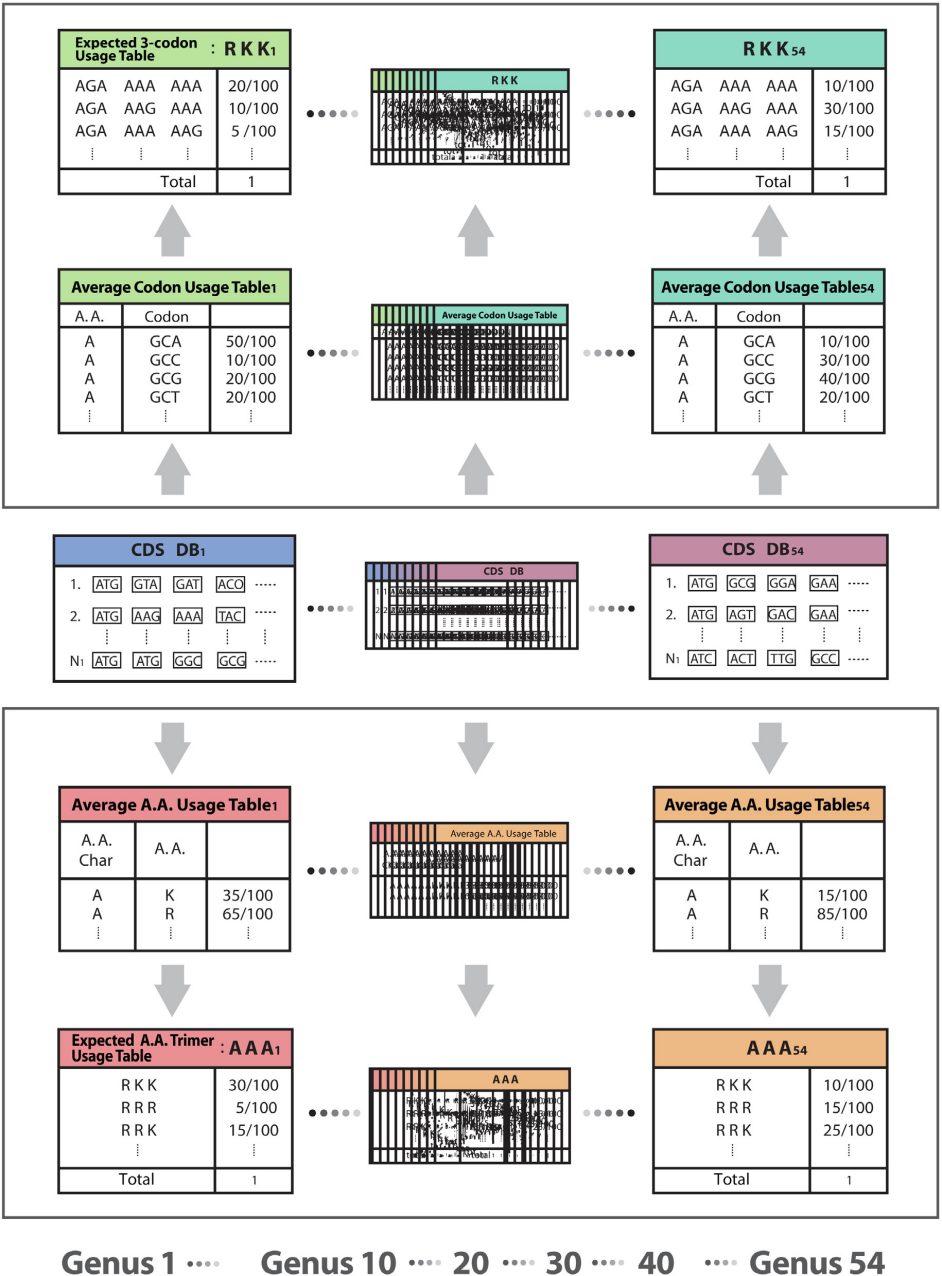
**Creation of expected usage tables for the rank probability scoring method** Average A.A. Usage Table and Average Codon Usage Table were calculated from the CDS database per genus. Expected A.A. Trimer Usage Tables and Expected 3-codon Usage Tables were created based on the Average A.A. Usage Table and the Average Codon Usage Table, respectively. All sequences and expected usage information in this figure are not real, but randomly chosen for illustration purposes only.

This method measures a standardized 3-codon usage relative to an expected 3-codon usage computed from three individual mono codon usages. The Average A.A. Usage Table (20 amino acids and stop codons for 12 A.A. Char monomers) and the Average Codon Usage Table (64 codons for 20 amino acid monomers and stop codons) were created based on CDS database per genus. 1296 Expected A.A. Trimer Usage Tables and 7674 Expected 3-codon Usage Tables were created based on the Average A.A. Usage Table and the Average Codon Usage Table, respectively (Figure 4).

A standardized 3-codon usage was calculated by dividing a 3-codon usage in a 3-codon Usage Table by an expected 3-codon usage in an Expected 3-codon Usage Table. Based on trimer usage biases and standardized 3-codon usages, we calculated a group mean for each taxon group and Kruskal Wallis (KW) test's h-score on ranks of 13 taxon groups, from which we developed a rank probability score per 3-codon DNA 9-mer. In addition to the trimer usage biases, the rank probability scoring method adds another set of 224,383 scores per genus into Genus-specific DB. This method is applicable only to CDS.

#### *P* value scoring method

We applied the concept of the sum of rolled numbers from a pair of dice to develop the *P* value scoring method (http://www.lucamoroni.it/the-dice-roll-sum-problem/). We drew analogies between the number of faces of a dice and 54 genera and between the number of dices we roll and the number of matching 3-codon DNA 9-mers identified in a reading frame of a query sequence. There were 54 possible ranks computed based on trimer usage biases per matching 3-codon DNA 9-mer. *P* value scores were calculated based on a sum of ranks of matching 3-codon DNA 9-mers. Computational costs of *P* values for all possible outcomes, sums of ranks, were too high. To reduce the computational costs, we approximated *P* values. We obtained sample data per number of matching 3-codon DNA 9-mers based on Equation (1).

(1) $$P\left(p,n,s\right)=\frac{1}{{\left(s\right)}^{n}}\sum_{k=0}^{]\left(p\;-\;n\right)/s[}{\left(-1\right)}^{k}\left(\begin{array}{c} n\\ K \end{array}\right)\left(\begin{array}{c} p-sk-1\\ n-1 \end{array}\right)$$

where *p* is the sum of ranks, *n* is the number of dices per roll, *s* is the number of faces of the dice, 54, and maximum of *k* is ](*p*-*n*)/*s*[ where ]*x*[ is the floor function (*e.g.*, ]7.9[ = 7). We created a table of *P* value scores per number of matching 3-codon DNA 9-mers. If a rank sum was less than one with the highest *P* value score, the approximate mean of all of the rank sums in each table, we multiplied the *P* value score with −1, indicating statistically non-significant outcome. In the test sets, the number of matching 3-codon DNA 9-mers varied widely, with a minimum of 30 and a maximum of 97. We have 624 tables in the *P* value score database covering 2–625 matching 3-codon DNA 9-mers.

### Implementation and program availability

SeSaMe was implemented using the Java programming language (Java 8). We provided two sets of the programs. One requires Apache commons math3 (3.3) and IO (2.4) libraries (www.apache.org), while the other does not. The programs consist of executable Java JAR files and Java class files for Linux/Unix operating systems. SeSaMe has been tested and confirmed to work on Linux systems — CentOS Linux 7 (www.centos.org) and is currently being used at the Biodiversity Center, Institut de Rechercheen Biologie Végétale, Département de Sciences Biologiques, Université de Montréal. The trimer usage probability scoring method offered to the public produces output of smaller size, but is sufficient for the purpose of taxonomic classification and is freely available at www.fungalsesame.org. There are no restrictions to use the programs by academic or non-academic organizations as long as they comply with the terms and conditions of the license agreements.

### Input, output, and options

SeSaMe utilizes a command-line interface. Input files should contain DNA sequence(s) in fasta format. The Java JAR files produce output files with sequence information (seq_id, matching A.A. Char Trimers, A.A. Trimers, and 3-codon DNA 9-mers) and genus information (rank, scores, and *P* value score). The output provides the information per reading frame per sequence. After processing the output file with Java class files, users are able to obtain a summary file containing one predicted outcome per query sequence. Java JAR files require users to give a mandatory command line argument — input file path. Java JAR files with the trimer probability scoring method may produce multiple genera as an answer if their scores have little difference. A user is given the option with 6 choices to select a cut-off value for the difference: 0.01, 0.05, 0.1, 0.15, 0.2, or 0.3. Users can give the option to the Java class file called compare_result_coding_non_coding.class. The default cut-off value is 0.05. The lower the cut-off value is, the fewer genera will be included in an answer.

## Results

We assessed the accuracy of the program by conducting classification experiments. We created metagenome test sets, ran the programs with them, and calculated the mean percentages of correct predictions. We showed the relationship between the correct prediction proportion and the *P* value score in order to provide users with useful examples in assessing the statistical significance of predicted outcomes.

### Metagenome test sets

We randomly chose 100 sequences from each of the CDS and non-CDS databases per genus. We randomly selected a starting base pair position in each of the randomly chosen sequences. From the starting position, we randomly selected an ending base pair position so that a sequence length is within the range of 150–300 bp. Both of the CDS and non-CDS test sets consisted of 4500 bacterial and 900 fungal sequences (including AMF).

### Mean percentages of correct predictions from the trimer usage probability scoring method

The mean percentages of correct predictions of the CDS and non-CDS test sets at the genus level were 71% and 50% for the bacterial group, 68% and 73% for the fungal group (excluding AMF), and 49% and 72% for AMF, respectively. AMF had the lowest prediction percentage in the CDS genus test sets possibly due to a large number of heterogeneous nuclei and horizontal gene transfers from a variety of endobacteria during their evolution [8], [10], [11], [12], [13], [14]. The mean percentages of correct predictions at the genus level and at higher taxonomic ranks of the 13 taxon groups are shown in Table 2.

**Table 2:** **Mean percentages of correct predictions at the rank of genus and at the higher ranks of 13 taxon groups**

	**Genus**	**CDS**		**Non-CDS**		**Genus**	**CDS**		**Non-CDS**	
		**Correct genus (%)**	**Correct taxon group (%)**	**Correct genus (%)**	**Correct taxon group (%)**		**Correct genus (%)**	**Correct taxon group (%)**	**Correct genus (%)**	**Correct taxon group (%)**
Bacteria	*Acidithiobacillus*	57	78	51	72	*Microbacterium*	87	96	60	89
	*Acidobacterium*	62	62	40	40	*Micrococcus*	93	97	48	89
	*Agrobacterium*	65	84	50	65	*Myxococcus*	88	89	27	39
	*Anabaena*	41	57	56	78	*Nitrobacter*	66	90	42	71
	*Azorhizobium*	87	97	49	80	*Nitrosococcus*	51	66	42	69
	*Azotobacter*	75	87	55	71	*Nitrosomonas*	45	45	33	33
	*Bacillus*	53	53	64	64	*Nitrosospira*	60	60	70	72
	*Bdellovibrio*	61	64	63	66	*Nocardia*	79	89	25	44
	*Beijerinckia*	65	83	56	66	*Nostoc*	58	60	62	68
	*Bradyrhizobium*	84	88	41	61	*Oscillatoria*	58	58	66	66
	*Caulobacter*	79	91	43	59	*Pseudanabaena*	76	77	48	53
	*Clostridium*	81	85	90	92	*Pseudomonas*	77	95	52	64
	*Cyanobacterium*	72	73	61	61	*Pseudonocardia*	88	96	29	74
	*Desulfotomaculum*	49	54	43	69	*Rhizobium*	70	81	48	60
	*Desulfovibrio*	43	52	52	55	*Rhodobacter*	85	94	32	66
	*Erwinia*	71	87	47	81	*Rickettsia*	68	68	67	67
	*Frankia*	72	90	18	50	*Shewanella*	75	83	83	86
	*Geobacter*	61	67	47	54	*Sinorhizobium*	67	83	51	69
	*Klebsiella*	79	95	59	77	*Sphingomonas*	76	92	31	71
	*Kocuria*	88	97	52	89	*Streptomyces*	89	96	55	56
	*Leuconostoc*	62	72	41	73	*Variovorax*	85	85	41	41
	*Mesorhizobium*	70	90	40	58	*Xanthomonas*	91	94	48	60
	*Methylococcus*	76	87	60	82					
	**Average accuracy**	**3185/4500**	**3587/4500**	**2238/4500**	**2970/4500**	**Mean percentage of correct predictions**	**71%**	**80%**	**50%**	**66%**
Fungi	AMF	49	49	72	72	*Oidiodendron*	68	68	71	71
	*Aspergillus*	72	72	77	77	*Phanerochaete*	52	67	66	91
	*Cenococcum*	54	66	88	90	*Scleroderma*	76	87	68	89
	*Cryptococcus*	72	87	78	89	*Sebacina*	58	89	66	90
	*Mycosphaerella*	88	93	69	81					
	**Average accuracy**	**589/900**	**678/900**	**655/900**	**750/900**	**Mean percentage of correct predictions**	**65%**	**75%**	**73%**	**83%**

*Note*: Average accuracy was calculated by dividing sum of correct predictions by total number of predictions made. For example, 3185 out of 4500 sequences were correctly classified to its taxonomic group at the rank of genus in testing bacterial CDS. Mean percentage of correct predictions was calculated by multiplying average accuracy by 100. For example, for bacterial CDS, (3185/4500) × 100 = 71. After genus in an answer was converted to a corresponding taxonomic group in the 13 taxon groups, the mean percentage of correct predictions (Correct taxon group %) was calculated. 13 taxon groups are indicated in the section, Scoring methods.

SeSaMe produced more than one genus as an answer per query sequence when multiple genera had little difference in their scores. We converted each predicted genus into one of the 13 taxon groups and calculated a proportion of the correct taxon group in answer per query sequence. We calculated the mean and the standard deviation of the proportions in each genus test set; 1 represented that answers contained correct taxon groups only, while 0 represented that answers contained incorrect taxon groups only (Tables S3 and S4). The means of the bacterial and fungal CDS test sets were 0.87 and 0.57, respectively (Tables S3 and S4).

**Supplementary figure S1:**
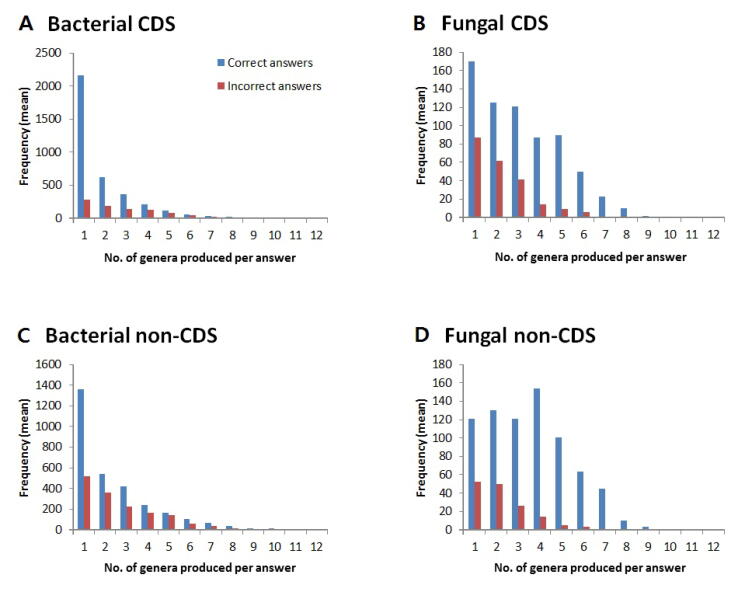
**Histogram of the number of genera produced per answer**. X-axis represents how many genera the trimer usage probability scoring method produced in an answer of a query sequence.

**Supplementary figure S2:**
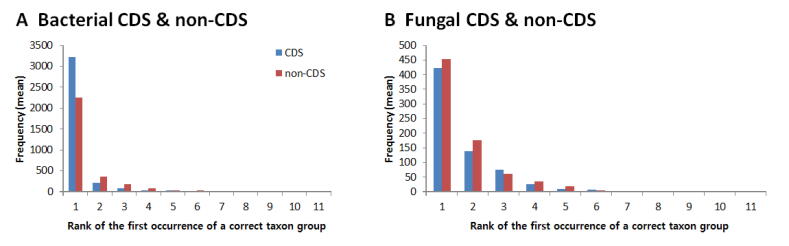
**Histogram of the rank of correct taxon group in answer**. X-axis represents at which rank a correct taxon group occurred for the first time in an answer of a query sequence.

**Supplementary figure S3:**
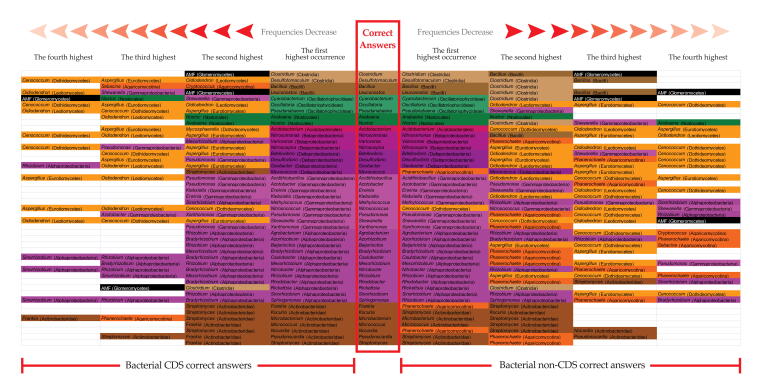
**Genera with similar trimer usage probability scores (Bacteria)**. The number of occurrences of each genus included in the correct answers was counted in the bacterial genus CDS and non-CDS test sets. The figure shows the genera with relatively high occurrences. In general, the genera that had little difference in their trimer usage probability scores belonged to the same phylum.

**Supplementary figure S4:**
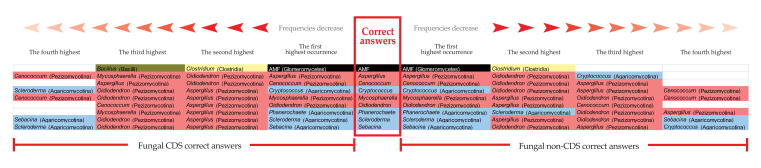
Genera with similar trimer usage probability scores (Fungi). The number of occurrence of each genus included in the correct answers was counted in the fungal genus CDS and non-CDS test sets. The figure shows the genera with relatively high occurrences. *Clostridium* occurred the second highest in the AMF test set based on trimer usage probability scoring method. Both Agaricomycotina and Pezizomycotina belong to Dikarya, and the former occurred the second highest in the answers of the latter, and vice versa.

60% and 46% of the correctly predicted sequences contained only one genus as an answer in the bacterial CDS and non-CDS test sets, respectively (Figure S1; Table S5). 90% and 76% of the correctly predicted sequences had a correct taxon group in the first rank in the bacterial CDS and non-CDS test sets, respectively (Figure S2; Table S6). Only 1%–5% of the sequences in the non-AMF test sets had AMF in an answer (Table S7). Although the trimer usage probability scoring method provides us not with the individual trimer usage biases but with result of multiplying all of the trimer usage biases, we can often derive general ideas about a query sequence from its answer (Figures S3 and S4). Does it contain only one genus in answer? Or what other genera does it contain in answer? For example, an AMF test sequence that contains *Clostridium* and AMF in answer may imply that the query sequence might have been acquired by horizontal gene transfer from a bacterium ancestor or that *Clostridium*’s ancestor might have become a heritable endosymbiont.

### Mean percentages of correct predictions from the rank probability scoring method

The mean percentages of correct predictions of the CDS test sets were 82% for the bacterial group, 72% for the fungal group (excluding AMF), and 42% for AMF. The mean percentages and the standard deviations of correct predictions of the CDS test sets were 64% ± 4.2%, 71% ± 6.4%, 84% ± 2.5%, 70% ± 2.8%, 73% ± 0%, 83% ± 8%, 74% ± 10%, 81% ± 7.8%, 88% ± 9.2%, 85% ± 5.9%, 42% ± 0%, 65% ± 6.4%, and 79% ± 6.7% for the 13 taxon groups. Compared to the trimer usage probability scoring method, the rank probability scoring method produced the higher mean and the smaller standard deviation for the bacterial group. In general, the rank probability scoring method showed improvement in performance. Although the means for Clostridia and Gammaproteobacteria were lower, their standard deviations were much smaller in the rank probability scoring method: 4.2% *vs.* 22% and 7.8% *vs.* 9.4%, respectively. The trimer usage probability scoring method showed better performance in Actinobacteria that had low within-group variation of trimer usage bias. In contrast, the rank probability scoring method showed better performance in genera that had relatively flat peakness in a frequency distribution curve of synonymous 3-codon DNA 9-mers, in addition to genera that had relatively large within-group variation of trimer usage bias.

### Relationship between correct prediction proportion and *P* value score

**Supplementary figure S5:**
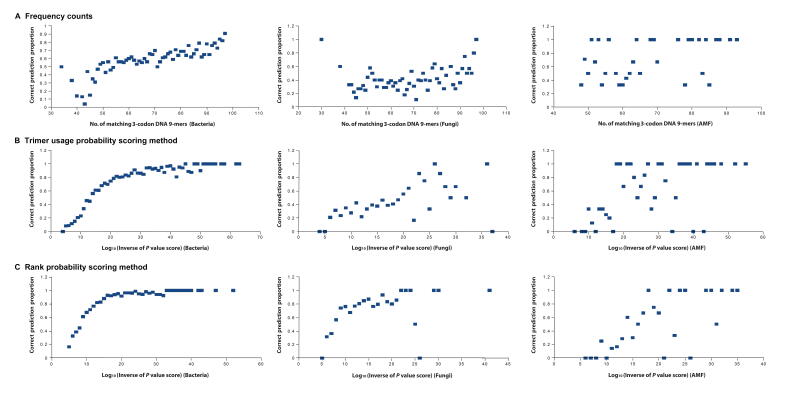
**Scatter plot of correct prediction proportion and (log10 (inverse of *P* value score))**. **A**. The mean of the correct prediction proportions was calculated per number of matching 3-codon DNA 9-mers in the bacterial, the fungal, and the AMF CDS test sets. For example, 22 sequences had 97 matching 3-codon DNA 9-mers and 20 of them were correctly classified in the bacterial test set; the mean was 0.91. **B**. and **C**. We calculated (log10 (inverse of *P* value score)) in order to show a relationship between the correct prediction proportion and *P* value score. For example, the multiplicative inverses of both *P* value scores, 1.0E-10 and 9.0E-10, were approximated to be 1.0E10, and base 10 logarithm of 1.0E10 was 10. **B** was based on the result from the trimer usage probability scoring method, while **C** was based on the result from the rank probability scoring method.

The means of the correct prediction proportions per number of matching 3-codon DNA 9-mers calculated based on the result of the trimer usage probability scoring method are shown in Figure S5A and Table S8. The means of correct prediction proportions per base 10 logarithm of an approximated inverse of *P* value score (log_10_ inverse of *P* value score) calculated based on result of the trimer usage probability scoring method and of the rank probability scoring method are shown in Figure S5B, Table S9, Figure S5C, and Table S10, respectively. We divided the results of each genus test set into quartiles and calculated the range of log_10_ inverse of *P* value score and the mean and the standard deviation of the correct prediction proportions in each quartile (Tables S11 and S12). The first ranked genus with the highest probability score always had positive *P* value score. In general, as log_10_ inverse of *P* value score became higher — *i.e.*, as positive *P* value score became lower — the correct prediction proportion increased in all test sets. The frequencies of fungal sequences that had a correct taxon group in the 1st, 2nd, 3rd, 4th, or 5th rank in an answer were comparable due to similarity of Dikarya (Figure S2; Table S6). Because the data for Figure S5B were generated based only on the first rank, the fungi showed relatively weak correlation between correct prediction proportion and log_10_ inverse of *P* value score.

### Classification of an example sequence

**Figure 5:**
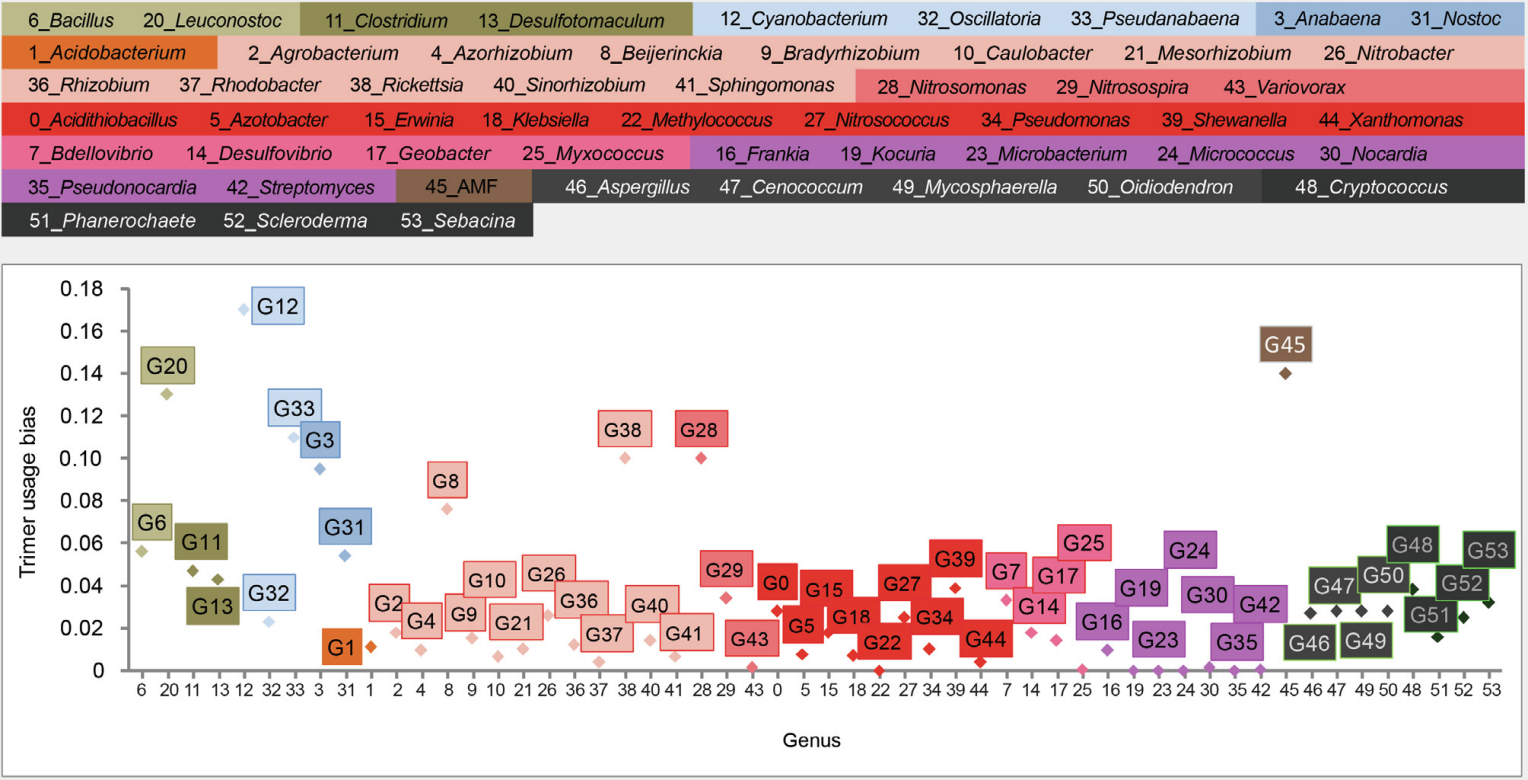
**Trimer usage biases of the 11th 3-codon DNA 9-mer, GATGATCAT, in 54 genera** Genera belonging to the same taxonomic group are indicated by the same background color. Because the program is zero-based, individual of 54 genera is labeled with a number from 0 to 53.

**Supplementary figure S6:**
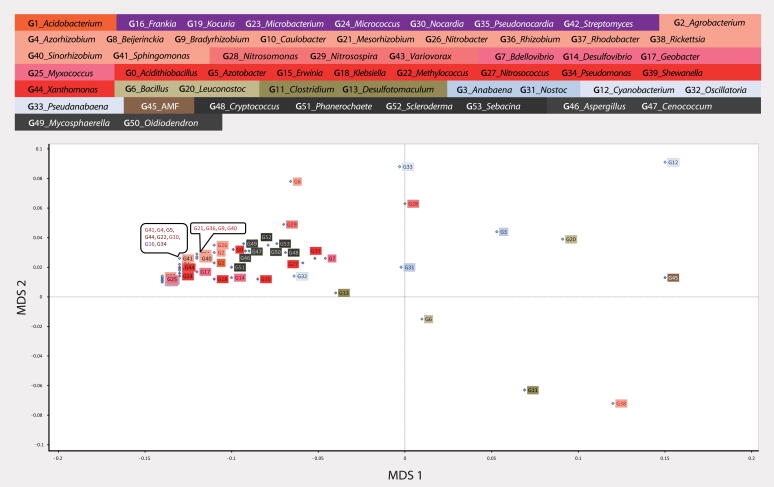
**Visualization of proximity relationships among 54 genera using MDS**. The XY axis graph represents proximity relationships among 54 genera based on trimer usage biases of 3-codon DNA 9-mers identified in the presumably open reading frame of the example sequence. Genera belonging to the same taxonomic group in 13 taxon groups are indicated by the same background color. Because the program is zero-based, individual of 54 genera is labeled with a number from 0 and to 53.

The example sequence (AAATCCCAATGTCAGAATAAAGAAACTACCAGATGATCATCCTGTTTATCCTGGGTATGGATTATTTGCTAACAAAGATCTTAAAAAATTTAATCTAGTCGTTTGTTATACTGGCAAAGTTACAAAAAGAGAAATTGGGGGTGAAGAAGGAAGTGA) was selected from the AMF CDS test set and is 156 bp in length. The sequence had the highest trimer usage probability score in the second reading frame in the forward direction, which was then assumed as the open reading frame. SeSaMe identified 49 matching 3-codon DNA 9-mers that were matched to Trimer Ref DB. The program correctly classified the example sequence into CDS of AMF. Firmicutes, Cyanobacteria, *Rickettsia*, and AMF had higher trimer usage biases than Proteobacteria, Actinobacteria, and Dikarya in a majority of 3-codon DNA 9-mers. Figure 5 shows trimer usage biases of the 11th 3-codon DNA 9-mer, GATGATCAT, in 54 genera. GATGATCAT belongs to A.A. Char Trimer, CCB and to A.A. Trimer, DDH. The multidimensional scaling (MDS) method was applied to a matrix containing trimer usage biases; it had 54 genera in rows and matching 3-codon DNA 9-mers identified in the open reading frame in columns (http://www.inf.uni-konstanz.de/algo/software/mdsj/) [34]. It visualized proximity relationships among 54 genera in XY axis graph (www.jfree.org). It showed that Actinobacteria, Alphaproteobacteria, and Dikarya were compactly clustered, while Betaproteobacteria were spread out in the left side of the graph (Figure S6). Nostocales, Oscillatoriophycideae, Bacilli, and Clostridia were scattered across in the right side. AMF, *Cyanobacterium*, and *Rickettsia* were located in the far-right side.

## Future work

Microorganisms contain a number of heterogeneous alternative sigma factors that are selectively induced in response to environmental stress [35]. They not only provide functionally specialized RNA polymerase subpopulations, but are also involved in regulating the expression of a set of target genes, or regulons [36], [37]. Ribosomal heterogeneity also has an important role in governing cellular stress. Sequence comparison of rRNA genes and ribosomal coding genes as well as sigma factor genes will be required in order to study their influence on adaptation of microorganisms [38], [39].

**Supplementary figure S7:**
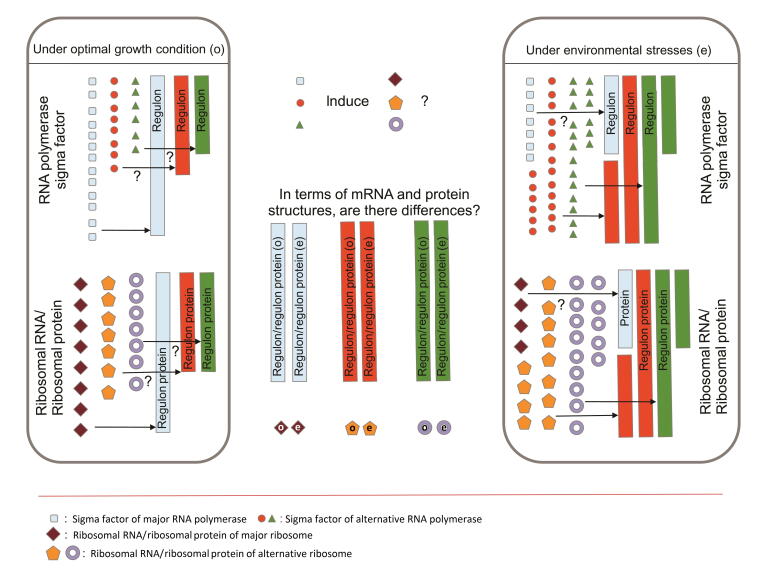
**Heterogeneous regulator subpopulations within a single isolate**. The symbols and question marks in the figure indicate the following questions from left to right. Under an optimal growth condition, do alternative regulators transcribe/translate genes? Does sigma factor regulate the expression of functionally specialized ribosomal rRNA and protein coding genes? Do heterogeneous regulator subpopulations produce structurally different gene products? Under environmental stress, do major regulators transcribe/translate genes?

Codon usage and codon context have been documented to play various important roles in microorganism’s adaptation to environment. If we sort the target genes in the CDS and in the non-CDS databases according to the alternative regulators, and create Genus-specific DB per group of the target genes, it may increase the accuracy of taxonomic classification. Moreover, comparative studies on alternative regulator subpopulations may provide useful insights into the development of genetic markers with which we can detect changes in microbial community structures in response to environmental stress (Figure S7). It may lead to new perspectives and strategies for improving the analysis of metagenome data, especially AMF inoculant field data sampled from highly stressful environments.

## Data availability

SeSaMe is freely available at www.fungalsesame.org.

## CRediT author statement

**Jee Eun Kang:** Conceptualization, Methodology, Software, Validation, Software, Writing - original draft. **Antonio Ciampi:** Supervision, Writing - review & editing. **Mohamed Hijri:** Supervision, Writing - review & editing. All authors read and approved the final manuscript.

## Competing interests

The authors have declared no competing interests.

## References

[1] Roy-Bolduc A., Hijri M. (2011). The use of mycorrhizae to enhance phosphorus uptake: a way out the phosphorus crisis. J Biofertil Biopestic.

[2] Hijri M. (2016). Analysis of a large dataset of mycorrhiza inoculation field trials on potato shows highly significant increases in yield. Mycorrhiza.

[3] Zarik L., Meddich A., Hijri M., Hafidi M., Ouhammou A., Ouahmane L. (2016). Use of arbuscular mycorrhizal fungi to improve the drought tolerance of *Cupressus atlantica G*. C R Biol.

[4] Hassan Sel-D, Bell TH, Stefani FO, Denis D, Hijri M, St-Arnaud M.Contrasting the community structure of arbuscular mycorrhizal fungi from hydrocarbon-contaminated and uncontaminated soils following willow (*Salix* spp. L.) planting. PLoS One 2014;9:e102838. 10.1371/journal.pone.0102838 PMC4102571 25032685

[5] Iffis B., St-Arnaud M., Hijri M. (2014). Bacteria associated with arbuscular mycorrhizal fungi within roots of plants growing in a soil highly contaminated with aliphatic and aromatic petroleum hydrocarbons. FEMS Microbiol Lett.

[6] de la Providencia I, Stéfani FO, Labridy M, St-Arnaud M, Hijri M. Arbuscular mycorrhizal fungal diversity associated with *Eleocharis obtusa* and *Panicum capillare* growing in an extreme petroleum hydrocarbon-polluted sedimentation basin. FEMS Microbiol Lett2015;362:fnv081. 10.1093/femsle/fnv081 25991810

[7] Chanda D., Sharma G.D., Jha D.K., Hijri M. (2014). Associations of arbuscular mycorrhizal (AM) fungi in the phytoremediation of trace metal (TM) contaminated soils. J Res Biol.

[8] Marleau J., Dalpe Y., St-Arnaud M., Hijri M. (2011). Spore development and nuclear inheritance in arbuscular mycorrhizal fungi. BMC Evol Biol.

[9] Boon E., Halary S., Bapteste E., Hijri M. (2015). Studying genome heterogeneity within the arbuscular mycorrhizal fungal cytoplasm. Genome Biol Evol.

[10] Hijri M., Redecker D., Petetot J.A.M.-C., Voigt K., Wöstemeyer J., Sanders I.R. (2002). Identification and isolation of two ascomycete fungi from spores of the arbuscular mycorrhizal fungus. Appl Environ Microbiol.

[11] Cruz A.F., Horii S., Ochiai S., Yasuda A., Ishii T. (2008). Isolation and analysis of bacteria associated with spores of *Gigaspora margarita*. J Appl Microbiol.

[12] Bonfante P. (2003). Plants, mycorrhizal fungi and endobacteria: a dialog among cells and genomes. Biol Bull.

[13] Naito M., Morton J.B., Pawlowska T.E. (2015). Minimal genomes of mycoplasma-related endobacteria are plastic and contain host-derived genes for sustained life within Glomeromycota. Proc Natl Acad Sci U S A.

[14] Torres-Cortés G., Ghignone S., Bonfante P., Schüßler A. (2015). Mosaic genome of endobacteria in arbuscular mycorrhizal fungi: transkingdom gene transfer in an ancient mycoplasma-fungus association. Proc Natl Acad Sci U S A.

[15] Tisserant E., Kohler A., Dozolme‐Seddas P., Balestrini R., Benabdellah K., Colard A. (2012). The transcriptome of the arbuscular mycorrhizal fungus *Glomus intraradices* (DAOM 197198) reveals functional tradeoffs in an obligate symbiont. New Phytol.

[16] Tisserant E., Malbreil M., Kuo A., Kohler A., Symeonidi A., Balestrini R. (2013). Genome of an arbuscular mycorrhizal fungus provides insight into the oldest plant symbiosis. Proc Natl Acad Sci U S A.

[17] Bécard G., Fortin J.A. (1988). Early events of vesicular-arbuscular mycorrhiza formation on Ri T-DNA transformed roots. New Phytol.

[18] Pride D.T., Meinersmann R.J., Wassenaar T.M., Blaser M.J. (2003). Evolutionary implications of microbial genome tetranucleotide frequency biases. Genome Res.

[19] Altschul S.F., Gish W., Miller W., Myers E.W., Lipman D.J. (1990). Basic local alignment search tool. J Mol Biol.

[20] Altschul S.F., Madden T.L., Schaffer A.A., Zhang J., Zhang Z., Miller W. (1997). Gapped BLAST and PSI-BLAST: a new generation of protein database search programs. Nucleic Acids Res.

[21] Rajendhran J., Gunasekaran P. (2011). Microbial phylogeny and diversity: small subunit ribosomal RNA sequence analysis and beyond. Microbiol Res.

[22] Akashi H. (1994). Synonymous codon usage in *Drosophila melanogaster*: natural selection and translational accuracy. Genetics.

[23] Gao F., Zhang C.T. (2004). Comparison of various algorithms for recognizing short coding sequences of human genes. Bioinformatics.

[24] Grantham R., Gautier C., Gouy M., Jacobzone M., Mercier R. (1981). Codon catalog usage is a genome strategy modulated for gene expressivity. Nucleic Acids Res.

[25] Karlin S., Mrázek J., Campbell A.M. (1997). Compositional biases of bacterial genomes and evolutionary implications. J Bacteriol.

[26] Sueoka N. (1962). On the genetic basis of variation and heterogeneity of DNA base composition. Proc Natl Acad Sci U S A.

[27] Kim M., Lee K.H., Yoon S.W., Kim B.S., Chun J., Yi H. (2013). Analytical tools and databases for metagenomics in the next-generation sequencing era. Genomics Inform.

[28] Jeffery S., Gardi C., Jones A., Montanarella L., Marmo L., Miko L. (2010). European atlas of soil biodiversity.

[29] Spain A.M., Krumholz L.R., Elshahed M.S. (2009). Abundance, composition, diversity and novelty of soil Proteobacteria. ISME J.

[30] Bonfante P., Anca I.A. (2009). Plants, mycorrhizal fungi, and bacteria: a network of interactions. Annu Rev Microbiol.

[31] Lecomte J., St-Arnaud M., Hijri M. (2011). Isolation and identification of soil bacteria growing at the expense of arbuscular mycorrhizal fungi. FEMS Microbiol Lett.

[32] Tedersoo L., Bahram M., Põlme S., Kõljalg U., Yorou N.S., Wijesundera R. (2014). Fungal biogeography. global diversity and geography of soil fungi. Science.

[33] Berman H.M., Westbrook J., Feng Z., Gilliland G., Bhat T.N., Weissig H. (2000). The protein data bank. Nucleic Acids Res.

[34] Algorithmics Group. MDSJ: Java library for multidimensional scaling (Version 0.2). [Internet]. Konstanz: University of Konstanz; 2009, http://www.inf.uni-konstanz.de/algo/software/mdsj/.

[35] Paget M.S. (2015). Bacterial sigma factors and anti-sigma factors: structure, function and distribution. Biomolecules.

[36] Zhang N., Buck M. (2015). A perspective on the enhancer dependent bacterial RNA polymerase. Biomolecules.

[37] Fisher M.A., Grimm D., Henion A.K., Elias A.F., Stewart P.E., Rosa P.A. (2005). *Borrelia burgdorferi* sigma54 is required for mammalian infection and vector transmission but not for tick colonization. Proc Natl Acad Sci U S A.

[38] Byrgazov K., Vesper O., Moll I. (2013). Ribosome heterogeneity: another level of complexity in bacterial translation regulation. Curr Opin Microbiol.

[39] Klappenbach J.A., Dunbar J.M., Schmidt T.M. (2000). rRNA operon copy number reflects ecological strategies of bacteria. Appl Environ Microbiol.

