# Supplementary material for: SeSaMe: Metagenome Sequence Classification of Arbuscular Mycorrhizal Fungi-associated Microorganisms
Source: Genomics Proteomics Bioinformatics. 2020 Dec 18;18(5):601–12. doi: 10.1016/j.gpb.2018.07.010 (PMC8377386; doi:10.1016/j.gpb.2018.07.010)
Supplement: Supplementary Table S1 [file mmc1.doc]

**Table S1 Total number of the bacterial genes per genus**

| **Bacterial genus** | **Total number of genes** | **Bacterial genus** | **Total number of genes** |
| --- | --- | --- | --- |
| *Acidithiobacillus* | 12,252 | *Microbacterium* | 3676 |
| *Acidobacterium* | 7924 | *Micrococcus* | 2236 |
| *Agrobacterium* | 22,773 | *Myxococcus* | 22,658 |
| *Anabaena* | 16,055 | *Nitrobacter* | 7448 |
| *Azorhizobium* | 4717 | *Nitrosococcus* | 9744 |
| *Azotobacter* | 15,105 | *Nitrosomonas* | 11,481 |
| *Bacillus* | 392,788 | *Nitrosospira* | 2805 |
| *Bdellovibrio* | 9939 | *Nocardia* | 19,851 |
| *Beijerinckia* | 3784 | *Nostoc* | 16,970 |
| *Bradyrhizobium* | 38,418 | *Oscillatoria* | 12,156 |
| *Caulobacter* | 17,132 | *Pseudanabaena* | 3854 |
| *Clostridium* | 168,122 | *Pseudomonas* | 321,496 |
| *Cyanobacterium* | 6268 | *Pseudonocardia* | 6797 |
| *Desulfotomaculum* | 21,547 | *Rhizobium* | 52,249 |
| *Desulfovibrio* | 51,438 | *Rhodobacter* | 20,963 |
| *Erwinia* | 31,931 | *Rickettsia* | 46,400 |
| *Frankia* | 29,711 | *Shewanella* | 102,711 |
| *Geobacter* | 34,729 | *Sinorhizobium* | 65,703 |
| *Klebsiella* | 69,640 | *Sphingomonas* | 15,561 |
| *Kocuria* | 2356 | *Streptomyces* | 149,826 |
| *Leuconostoc* | 16,970 | *Variovorax* | 18,984 |
| *Mesorhizobium* | 30,388 | *Xanthomonas* | 65,650 |
| *Methylococcus* | 2960 |  |  |
