# Supplementary material for: SeSaMe: Metagenome Sequence Classification of Arbuscular Mycorrhizal Fungi-associated Microorganisms
Source: Genomics Proteomics Bioinformatics. 2020 Dec 18;18(5):601–12. doi: 10.1016/j.gpb.2018.07.010 (PMC8377386; doi:10.1016/j.gpb.2018.07.010)
Supplement: Supplementary Table S2 [file mmc2.doc]

**Table S2 Total number of the fungal genes and introns per genus**

| **Fungal genus** | **Total number of genes** | **Total number of introns** |
| --- | --- | --- |
| AMF | 21,929 | 52,385 |
| *Aspergillus* | 19,695 | 38,513 |
| *Cenococcum* | 14,748 | 27,036 |
| *Cryptococcus* | 13,174 | 68,068 |
| *Mycosphaerella* | 13,107 | 33,856 |
| *Oidiodendron* | 16,703 | 32,542 |
| *Phanerochaete* | 10,048 | 48,688 |
| *Scleroderma* | 21,012 | 65,184 |
| *Sebacina* | 15,312 | 58,256 |
