# Supplementary material for: SeSaMe: Metagenome Sequence Classification of Arbuscular Mycorrhizal Fungi-associated Microorganisms
Source: Genomics Proteomics Bioinformatics. 2020 Dec 18;18(5):601–12. doi: 10.1016/j.gpb.2018.07.010 (PMC8377386; doi:10.1016/j.gpb.2018.07.010)
Supplement: Supplementary Table S3 [file mmc3.doc]

**Table S3 Correct taxon group proportion in an answer in the** bacterial test sets

| **Genus** | **CDS** | |  | **Non-CDS** | | **Genus** | **CDS** | |  | **Non-CDS** | |
| --- | --- | --- | --- | --- | --- | --- | --- | --- | --- | --- | --- |
|  | Mean | SD |  | Mean | SD |  | Mean | SD |  | Mean | SD |
| *Acidithiobacillus* | 0.691 | 0.327 |  | 0.552 | 0.344 | *Microbacterium* | 0.948 | 0.166 |  | 0.892 | 0.229 |
| *Acidobacterium* | 0.825 | 0.303 |  | 0.616 | 0.379 | *Micrococcus* | 0.979 | 0.096 |  | 0.857 | 0.261 |
| *Agrobacterium* | 0.860 | 0.236 |  | 0.646 | 0.317 | *Myxococcus* | 0.912 | 0.216 |  | 0.562 | 0.317 |
| *Anabaena* | 0.762 | 0.311 |  | 0.745 | 0.299 | *Nitrobacter* | 0.911 | 0.187 |  | 0.767 | 0.295 |
| *Azorhizobium* | 0.922 | 0.188 |  | 0.708 | 0.294 | *Nitrosococcus* | 0.632 | 0.344 |  | 0.577 | 0.351 |
| *Azotobacter* | 0.788 | 0.287 |  | 0.792 | 0.298 | *Nitrosomonas* | 0.776 | 0.343 |  | 0.719 | 0.384 |
| *Bacillus* | 0.758 | 0.334 |  | 0.610 | 0.322 | *Nitrosospira* | 0.826 | 0.297 |  | 0.722 | 0.351 |
| *Bdellovibrio* | 0.886 | 0.247 |  | 0.715 | 0.358 | *Nocardia* | 0.850 | 0.254 |  | 0.755 | 0.285 |
| *Beijerinckia* | 0.834 | 0.279 |  | 0.662 | 0.341 | *Nostoc* | 0.927 | 0.169 |  | 0.770 | 0.305 |
| *Bradyrhizobium* | 0.928 | 0.175 |  | 0.696 | 0.289 | *Oscillatoria* | 0.830 | 0.297 |  | 0.696 | 0.357 |
| *Caulobacter* | 0.897 | 0.203 |  | 0.815 | 0.269 | *Pseudanabaena* | 0.939 | 0.184 |  | 0.643 | 0.344 |
| *Clostridium* | 0.864 | 0.252 |  | 0.792 | 0.285 | *Pseudomonas* | 0.840 | 0.250 |  | 0.664 | 0.327 |
| *Cyanobacterium* | 0.960 | 0.150 |  | 0.736 | 0.328 | *Pseudonocardia* | 0.972 | 0.127 |  | 0.911 | 0.202 |
| *Desulfotomaculum* | 0.782 | 0.308 |  | 0.662 | 0.304 | *Rhizobium* | 0.896 | 0.191 |  | 0.673 | 0.336 |
| *Desulfovibrio* | 0.592 | 0.330 |  | 0.504 | 0.317 | *Rhodobacter* | 0.934 | 0.190 |  | 0.687 | 0.267 |
| *Erwinia* | 0.841 | 0.279 |  | 0.585 | 0.339 | *Rickettsia* | 0.904 | 0.234 |  | 0.823 | 0.290 |
| *Frankia* | 0.907 | 0.201 |  | 0.841 | 0.277 | *Shewanella* | 0.773 | 0.336 |  | 0.715 | 0.337 |
| *Geobacter* | 0.770 | 0.299 |  | 0.594 | 0.344 | *Sinorhizobium* | 0.855 | 0.239 |  | 0.776 | 0.304 |
| *Klebsiella* | 0.893 | 0.236 |  | 0.746 | 0.331 | *Sphingomonas* | 0.917 | 0.181 |  | 0.757 | 0.267 |
| *Kocuria* | 0.967 | 0.130 |  | 0.885 | 0.238 | *Streptomyces* | 0.953 | 0.158 |  | 0.848 | 0.262 |
| *Leuconostoc* | 0.838 | 0.282 |  | 0.594 | 0.296 | *Variovorax* | 0.938 | 0.195 |  | 0.684 | 0.337 |
| *Mesorhizobium* | 0.876 | 0.214 |  | 0.729 | 0.304 | *Xanthomonas* | 0.904 | 0.215 |  | 0.751 | 0.319 |
| *Methylococcus* | 0.796 | 0.289 |  | 0.698 | 0.346 | **Mean** | **0.87** | **0.25** |  | **0.72** | **0.32** |

*Note*: After genera in an answer were converted to the 13 taxon groups, the proportion of the correct taxon group was calculated per sequence in the genus test set. The mean and the standard deviation of the proportions of the correct taxon group are shown in the table.
