# Supplementary material for: SeSaMe: Metagenome Sequence Classification of Arbuscular Mycorrhizal Fungi-associated Microorganisms
Source: Genomics Proteomics Bioinformatics. 2020 Dec 18;18(5):601–12. doi: 10.1016/j.gpb.2018.07.010 (PMC8377386; doi:10.1016/j.gpb.2018.07.010)
Supplement: Supplementary Table S5 [file mmc5.doc]

**Table S5 Frequency of the number of genera produced per answer**

|  | **1** | **2** | **3** | **4** | **5** | **6** | **7** | **8** | **9** | **10** | **11** | **12** | **Sum** |
| --- | --- | --- | --- | --- | --- | --- | --- | --- | --- | --- | --- | --- | --- |
| Bact. CDS: correct | 2160 | 627 | 361 | 210 | 115 | 56 | 33 | 17 | 2 | 4 | 1 | 1 | 3587 |
| Bact. CDS: incorrect | 281 | 189 | 143 | 126 | 80 | 49 | 23 | 16 | 6 | 0 | 0 | 0 | 913 |
| Fung. CDS: correct | 170 | 125 | 121 | 87 | 90 | 50 | 23 | 10 | 2 | 0 | 0 | 0 | 678 |
| Fung. CDS: incorrect | 87 | 62 | 41 | 14 | 9 | 6 | 1 | 1 | 0 | 1 | 0 | 0 | 222 |
|  |  |  |  |  |  |  |  |  |  |  |  | **Total** | 5400 |
| Bact. non-CDS: correct | 1358 | 539 | 418 | 242 | 163 | 108 | 67 | 37 | 18 | 14 | 5 | 1 | 2970 |
| Bact. non-CDS: incorrect | 521 | 363 | 222 | 165 | 144 | 58 | 38 | 15 | 3 | 0 | 1 | 0 | 1530 |
| Fung. non-CDS: correct | 121 | 130 | 121 | 154 | 101 | 63 | 45 | 10 | 3 | 1 | 1 | 0 | 750 |
| Fung. non-CDS: incorrect | 52 | 50 | 26 | 14 | 5 | 3 | 0 | 0 | 0 | 0 | 0 | 0 | 150 |
|  |  |  |  |  |  |  |  |  |  |  |  | **Total** | 5400 |

*Note*: The table shows the frequencies of how many genera the trimer usage probability scoring method produced in an answer of a query sequence in the correct and the incorrect results in the bacterial (bact.) and the fungal (fung.) CDS and non-CDS test sets. Data for Figure S1.
