# Supplementary material for: SeSaMe: Metagenome Sequence Classification of Arbuscular Mycorrhizal Fungi-associated Microorganisms
Source: Genomics Proteomics Bioinformatics. 2020 Dec 18;18(5):601–12. doi: 10.1016/j.gpb.2018.07.010 (PMC8377386; doi:10.1016/j.gpb.2018.07.010)
Supplement: Supplementary Table S6 [file mmc6.doc]

**Table S6 Frequency of the rank of correct taxon group in answer**

|  | **0** | **1** | **2** | **3** | **4** | **5** | **6** | **7** | **8** | **9** | **10** | **Sum** | **Total** |
| --- | --- | --- | --- | --- | --- | --- | --- | --- | --- | --- | --- | --- | --- |
| Bact. CDS | 3218 | 208 | 86 | 33 | 29 | 9 | 1 | 3 | 0 | 0 | 0 | 3587 | 4500 |
| Bact. non-CDS | 2260 | 368 | 178 | 75 | 38 | 28 | 9 | 5 | 5 | 2 | 2 | 2970 | 4500 |
| Fung. CDS | 422 | 138 | 76 | 25 | 10 | 6 | 0 | 1 | 0 | 0 | 0 | 678 | 900 |
| Fung. non-CDS | 453 | 177 | 60 | 36 | 19 | 5 | 0 | 0 | 0 | 0 | 0 | 750 | 900 |

*Note*: The table shows the frequency of at which rank the trimer usage probability scoring method produced a correct taxon group for the first time in an answer of a query sequence in the correct results in the bacterial (bact.) and the fungal (fung.) CDS and non-CDS test sets. Data for Figure S2.
