# Supplementary material for: SeSaMe: Metagenome Sequence Classification of Arbuscular Mycorrhizal Fungi-associated Microorganisms
Source: Genomics Proteomics Bioinformatics. 2020 Dec 18;18(5):601–12. doi: 10.1016/j.gpb.2018.07.010 (PMC8377386; doi:10.1016/j.gpb.2018.07.010)
Supplement: Supplementary Table S7 [file mmc7.doc]

**Table S7 Percentage of the bacterial, fungal, and AMF groups in answers**

|  | **Correct prediction**  **percentages (genus)** | **The other group in answers** | **AMF in answers** |
| --- | --- | --- | --- |
| Bact. CDS | 3185/4500: 71% | 197/3185: 6% | 119/4500: 3% |
| Bact. non-CDS | 2238/4500: 50% | 489/2238: 22% | 229/4500: 5% |
| Fung. CDS | 589/900: 65% | 93/589: 16% | 6/800: 1% |
| Fung. non-CDS | 655/900: 73% | 157/655: 24% | 10/800: 1% |
| AMF CDS | 49/100: 49% | 24/49: 49% |  |
| AMF non-CDS | 72/100: 72% | 35/72: 49% |  |

*Note*: The column, The other group in answers, indicates the percentage of the fungal (fung.) and the bacterial (bact.) group in answers of the bacterial and the fungal test sets, respectively, while it indicates the percentage of the bacterial group in case of the AMF test sets. The column, AMF in answers, indicates the percentage of AMF in answers of the bacterial and the fungal test sets.
