# Supplementary material for: SeSaMe: Metagenome Sequence Classification of Arbuscular Mycorrhizal Fungi-associated Microorganisms
Source: Genomics Proteomics Bioinformatics. 2020 Dec 18;18(5):601–12. doi: 10.1016/j.gpb.2018.07.010 (PMC8377386; doi:10.1016/j.gpb.2018.07.010)
Supplement: Supplementary Table S8 [file mmc8.doc]

**Table S8 Correlation between the correct prediction proportion and the number of matching 3-codon DNA 9-mers**

| **Bacteria** | |  | **Fungi** | |  | **AMF** | |
| --- | --- | --- | --- | --- | --- | --- | --- |
| **No. of**  **matching**  **3-codon**  **DNA 9-mers** | **Correct**  **prediction**  **proportion** |  | **No. of**  **matching**  **3-codon**  **DNA 9-mers** | **Correct**  **prediction**  **proportion** |  | **No. of**  **matching**  **3-codon**  **DNA 9-mers** | **Correct**  **prediction**  **proportion** |
| 34 | 0.5 |  | 30 | 1 |  | 48 | 0.33 |
| 38 | 0.33 |  | 38 | 0.6 |  | 49 | 0.71 |
| 40 | 0.14 |  | 42 | 0.33 |  | 50 | 0.5 |
| 42 | 0.13 |  | 43 | 0.33 |  | 51 | 1 |
| 43 | 0.04 |  | 44 | 0.22 |  | 52 | 0.67 |
| 44 | 0.44 |  | 45 | 0.14 |  | 53 | 1 |
| 45 | 0.15 |  | 46 | 0.27 |  | 54 | 0.33 |
| 46 | 0.35 |  | 47 | 0.27 |  | 55 | 0.5 |
| 47 | 0.31 |  | 48 | 0.32 |  | 56 | 1 |
| 48 | 0.47 |  | 49 | 0.25 |  | 58 | 0.5 |
| 49 | 0.53 |  | 50 | 0.44 |  | 59 | 0.33 |
| 50 | 0.55 |  | 51 | 0.58 |  | 60 | 0.33 |
| 51 | 0.43 |  | 52 | 0.5 |  | 61 | 0.43 |
| 52 | 0.56 |  | 53 | 0.4 |  | 62 | 0.5 |
| 53 | 0.46 |  | 54 | 0.3 |  | 63 | 0.67 |
| 54 | 0.49 |  | 55 | 0.4 |  | 64 | 1 |
| 55 | 0.61 |  | 56 | 0.4 |  | 65 | 0.5 |
| 56 | 0.56 |  | 57 | 0.29 |  | 68 | 1 |
| 57 | 0.56 |  | 58 | 0.29 |  | 69 | 1 |
| 58 | 0.55 |  | 59 | 0.36 |  | 70 | 0.67 |
| 59 | 0.58 |  | 60 | 0.39 |  | 76 | 1 |
| 60 | 0.6 |  | 61 | 0.31 |  | 78 | 0.33 |
| 61 | 0.62 |  | 62 | 0.36 |  | 79 | 1 |
| 62 | 0.58 |  | 63 | 0.25 |  | 80 | 1 |
| 63 | 0.53 |  | 64 | 0.39 |  | 82 | 1 |
| 64 | 0.57 |  | 65 | 0.42 |  | 83 | 0.5 |
| 65 | 0.55 |  | 66 | 0.18 |  | 84 | 1 |
| 66 | 0.6 |  | 67 | 0.26 |  | 85 | 0.33 |
| 67 | 0.56 |  | 68 | 0.35 |  | 87 | 1 |
| 68 | 0.66 |  | 69 | 0.53 |  | 88 | 1 |
| 69 | 0.65 |  | 70 | 0.31 |  | 91 | 1 |
| 70 | 0.7 |  | 71 | 0.11 |  | 93 | 1 |
| 71 | 0.5 |  | 72 | 0.4 |  |  |  |
| 72 | 0.56 |  | 73 | 0.39 |  |  |  |
| 73 | 0.61 |  | 74 | 0.5 |  |  |  |
| 74 | 0.62 |  | 75 | 0.4 |  |  |  |
| 75 | 0.66 |  | 76 | 0.25 |  |  |  |
| 76 | 0.68 |  | 77 | 0.39 |  |  |  |
| 77 | 0.59 |  | 78 | 0.58 |  |  |  |
| 78 | 0.71 |  | 79 | 0.64 |  |  |  |
| 79 | 0.64 |  | 80 | 0.42 |  |  |  |
| 80 | 0.69 |  | 81 | 0.36 |  |  |  |
| 81 | 0.69 |  | 82 | 0.57 |  |  |  |
| 82 | 0.64 |  | 83 | 0.27 |  |  |  |
| 83 | 0.76 |  | 84 | 0.47 |  |  |  |
| 84 | 0.62 |  | 86 | 0.6 |  |  |  |
| 85 | 0.66 |  | 87 | 0.33 |  |  |  |
| 86 | 0.71 |  | 88 | 0.27 |  |  |  |
| 87 | 0.79 |  | 89 | 0.5 |  |  |  |
| 88 | 0.62 |  | 90 | 0.36 |  |  |  |
| 89 | 0.65 |  | 91 | 0.57 |  |  |  |
| 90 | 0.78 |  | 92 | 0.75 |  |  |  |
| 91 | 0.65 |  | 93 | 0.5 |  |  |  |
| 92 | 0.76 |  | 94 | 0.57 |  |  |  |
| 93 | 0.79 |  | 95 | 0.5 |  |  |  |
| 94 | 0.73 |  | 96 | 0.8 |  |  |  |
| 95 | 0.84 |  | 97 | 1 |  |  |  |
| 96 | 0.82 |  |  |  |  |  |  |
| 97 | 0.91 |  |  |  |  |  |  |

*Note*: Data for Figure S5A.
