# Supplementary material for: SeSaMe: Metagenome Sequence Classification of Arbuscular Mycorrhizal Fungi-associated Microorganisms
Source: Genomics Proteomics Bioinformatics. 2020 Dec 18;18(5):601–12. doi: 10.1016/j.gpb.2018.07.010 (PMC8377386; doi:10.1016/j.gpb.2018.07.010)
Supplement: Supplementary Table S10 [file mmc10.doc]

**Table S10 Correlation between the correct prediction proportion of the rank probability scoring method and *P* value score**

| **Bacteria** | |  | **Fungi** | |  | **AMF** | |
| --- | --- | --- | --- | --- | --- | --- | --- |
| **Log_10_**  **(inverse of**  ***P* value score)** | **Correct prediction**  **proportion** |  | **Log_10_**  **(inverse of**  ***P* value score)** | **Correct**  **prediction**  **proportion** |  | **Log_10_**  **(inverse of**  ***P* value score)** | **Correct**  **prediction**  **proportion** |
| 5 | 0.166 |  | 5 | 0 |  | 6 | 0 |
| 6 | 0.325 |  | 6 | 0.315 |  | 7 | 0 |
| 7 | 0.386 |  | 7 | 0.363 |  | 8 | 0 |
| 8 | 0.445 |  | 8 | 0.565 |  | 9 | 0.25 |
| 9 | 0.612 |  | 9 | 0.74 |  | 10 | 0 |
| 10 | 0.676 |  | 10 | 0.762 |  | 11 | 0.142 |
| 11 | 0.717 |  | 11 | 0.674 |  | 12 | 0.166 |
| 12 | 0.768 |  | 12 | 0.767 |  | 13 | 0.285 |
| 13 | 0.821 |  | 13 | 0.805 |  | 14 | 0.6 |
| 14 | 0.828 |  | 14 | 0.847 |  | 15 | 0.3 |
| 15 | 0.881 |  | 15 | 0.871 |  | 16 | 0.5 |
| 16 | 0.929 |  | 16 | 0.763 |  | 17 | 0.666 |
| 17 | 0.922 |  | 17 | 0.794 |  | 18 | 1 |
| 18 | 0.939 |  | 18 | 0.933 |  | 19 | 0.75 |
| 19 | 0.952 |  | 19 | 0.833 |  | 20 | 0.666 |
| 20 | 0.916 |  | 20 | 0.8 |  | 21 | 0 |
| 21 | 0.964 |  | 21 | 0.857 |  | 22 | 1 |
| 22 | 0.965 |  | 22 | 1 |  | 23 | 0.333 |
| 23 | 0.961 |  | 23 | 1 |  | 24 | 1 |
| 24 | 0.989 |  | 24 | 1 |  | 25 | 1 |
| 25 | 0.95 |  | 25 | 0.5 |  | 26 | 0 |
| 26 | 0.942 |  | 26 | 0 |  | 29 | 1 |
| 27 | 0.98 |  | 29 | 1 |  | 30 | 1 |
| 28 | 0.96 |  | 30 | 1 |  | 31 | 0.5 |
| 29 | 0.975 |  | 41 | 1 |  | 32 | 1 |
| 30 | 0.941 |  |  |  |  | 34 | 1 |
| 31 | 0.941 |  |  |  |  | 35 | 1 |
| 32 | 0.923 |  |  |  |  |  |  |
| 33 | 1 |  |  |  |  |  |  |
| 34 | 1 |  |  |  |  |  |  |
| 35 | 1 |  |  |  |  |  |  |
| 36 | 1 |  |  |  |  |  |  |
| 37 | 1 |  |  |  |  |  |  |
| 38 | 1 |  |  |  |  |  |  |
| 39 | 1 |  |  |  |  |  |  |
| 40 | 1 |  |  |  |  |  |  |
| 42 | 1 |  |  |  |  |  |  |
| 43 | 1 |  |  |  |  |  |  |
| 47 | 1 |  |  |  |  |  |  |
| 52 | 1 |  |  |  |  |  |  |

*Note*: The mean of the correct prediction proportions per (log10 (inverse of *P* value score)) was calculated based on the first ranked genus with the highest probability score in the result from the rank probability scoring method applied to the bacterial, the fungal, and the AMF CDS test sets. Data for Figure S5C.
