# Supplementary material for: SeSaMe: Metagenome Sequence Classification of Arbuscular Mycorrhizal Fungi-associated Microorganisms
Source: Genomics Proteomics Bioinformatics. 2020 Dec 18;18(5):601–12. doi: 10.1016/j.gpb.2018.07.010 (PMC8377386; doi:10.1016/j.gpb.2018.07.010)
Supplement: Supplementary Table S11 [file mmc11.doc]

**Table S11 Relationship between the correct prediction proportion of the trimer usage probability scoring method and *P* value score in quartiles**

| **Genus** | **0–25th percentile** | | |  | **26–50th percentile** | | |  | **51–75th percentile** | | |  | **76–100th percentile** | | |
| --- | --- | --- | --- | --- | --- | --- | --- | --- | --- | --- | --- | --- | --- | --- | --- |
|  | **Range** | **Mean** | **SD** |  | **Range** | **Mean** | **SD** |  | **Range** | **Mean** | **SD** |  | **Range** | **Mean** | **SD** |
| *Acidithiobacillus* | 5**–**8 | 0.151 | 0.18 |  | 9**–**13 | 0.41 | 0.217 |  | 14**–**19 | 0.461 | 0.402 |  | 20**–**32 | 0.6 | 0.547 |
| *Acidobacterium* | 6**–**10 | 0.276 | 0.18 |  | 11**–**15 | 0.564 | 0.223 |  | 16**–**20 | 0.62 | 0.073 |  | 21**–**28 | 0.875 | 0.306 |
| *Agrobacterium* | 4**–**9 | 0.045 | 0.0622 |  | 10**–**14 | 0.425 | 0.139 |  | 15**–**19 | 0.733 | 0.278 |  | 20**–**30 | 0.444 | 0.455 |
| *Anabaena* | 5**–**13 | 0.157 | 0.329 |  | 14**–**22 | 0.327 | 0.321 |  | 23**–**32 | 0.529 | 0.425 |  | 33**–**51 | 0.85 | 0.337 |
| *Azorhizobium* | 7**–**15 | 0.333 | 0.388 |  | 16**–**23 | 0.75 | 0.277 |  | 24**–**31 | 0.975 | 0.0707 |  | 32**–**43 | 1 | 0 |
| *Azotobacter* | 6**–**12 | 0.276 | 0.34 |  | 13**–**20 | 0.783 | 0.357 |  | 21**–**27 | 0.959 | 0.107 |  | 28**–**41 | 1 | 0 |
| *Bacillus* | 5**–**12 | 0.182 | 0.227 |  | 13**–**21 | 0.416 | 0.333 |  | 22**–**30 | 0.679 | 0.22 |  | 31**–**47 | 0.833 | 0.25 |
| *Bdellovibrio* | 6**–**11 | 0.205 | 0.186 |  | 12**–**18 | 0.669 | 0.308 |  | 19**–**25 | 0.821 | 0.144 |  | 26**–**37 | 0.928 | 0.188 |
| *Beijerinckia* | 5**–**9 | 0.08 | 0.109 |  | 10**–**15 | 0.473 | 0.279 |  | 16**–**21 | 0.906 | 0.0924 |  | 22**–**34 | 1 | 0 |
| *Bradyrhizobium* | 4**–**11 | 0.178 | 0.237 |  | 12**–**19 | 0.667 | 0.237 |  | 20**–**26 | 0.857 | 0.196 |  | 27**–**40 | 0.937 | 0.176 |
| *Caulobacter* | 6**–**13 | 0.328 | 0.37 |  | 14**–**22 | 0.781 | 0.213 |  | 23**–**31 | 0.982 | 0.0505 |  | 32**–**42 | 1 | 0 |
| *Clostridium* | 6**–**20 | 0.424 | 0.389 |  | 21**–**32 | 0.83 | 0.211 |  | 33**–**45 | 1 | 0 |  | 47**–**63 | 0.972 | 0.0962 |
| *Cyanobacterium* | 7**–**19 | 0.233 | 0.344 |  | 20**–**30 | 0.903 | 0.205 |  | 31**–**41 | 0.893 | 0.238 |  | 42**–**54 | 1 | 0 |
| *Desulfotomaculum* | 5**–**10 | 0.201 | 0.178 |  | 11**–**16 | 0.42 | 0.291 |  | 17**–**22 | 0.48 | 0.43 |  | 23**–**38 | 0.69 | 0.365 |
| *Desulfovibrio* | 6**–**10 | 0.253 | 0.31 |  | 11**–**15 | 0.276 | 0.145 |  | 16**–**20 | 0.23 | 0.338 |  | 21**–**30 | 0.0833 | 0.204 |
| *Erwinia* | 5**–**10 | 0.163 | 0.146 |  | 11**–**16 | 0.488 | 0.289 |  | 17**–**22 | 0.877 | 0.142 |  | 23**–**34 | 1 | 0 |
| *Frankia* | 5**–**12 | 0.272 | 0.309 |  | 13**–**20 | 0.72 | 0.197 |  | 21**–**27 | 0.45 | 0.326 |  | 28**–**46 | 0.375 | 0.443 |
| *Geobacter* | 6**–**9 | 0.243 | 0.204 |  | 10**–**14 | 0.464 | 0.232 |  | 15**–**18 | 0.69 | 0.359 |  | 19**–**28 | 0.85 | 0.223 |
| *Klebsiella* | 5**–**10 | 0.236 | 0.409 |  | 11**–**17 | 0.642 | 0.135 |  | 18**–**23 | 0.979 | 0.051 |  | 24**–**32 | 1 | 0 |
| *Kocuria* | 6**–**15 | 0.275 | 0.415 |  | 17**–**27 | 0.75 | 0.403 |  | 29**–**38 | 0.987 | 0.0395 |  | 39**–**54 | 0.977 | 0.0753 |
| *Leuconostoc* | 7**–**14 | 0.166 | 0.288 |  | 15**–**23 | 0.854 | 0.242 |  | 24**–**31 | 0.979 | 0.0589 |  | 32**–**44 | 1 | 0 |
| *Mesorhizobium* | 5**–**10 | 0.116 | 0.139 |  | 11**–**16 | 0.546 | 0.345 |  | 17**–**22 | 0.682 | 0.205 |  | 23**–**31 | 0.597 | 0.395 |
| *Methylococcus* | 6**–**10 | 0.14 | 0.219 |  | 11**–**15 | 0.711 | 0.309 |  | 16**–**20 | 0.763 | 0.152 |  | 21**–**30 | 0.934 | 0.106 |
| *Microbacterium* | 7**–**16 | 0.0937 | 0.265 |  | 17**–**26 | 0.922 | 0.171 |  | 27**–**34 | 0.933 | 0.128 |  | 37**–**50 | 1 | 0 |
| *Micrococcus* | 10**–**19 | 0.15 | 0.253 |  | 20**–**30 | 0.91 | 0.156 |  | 31**–**40 | 0.983 | 0.0527 |  | 41**–**55 | 1 | 0 |
| *Myxococcus* | 6**–**16 | 0.461 | 0.373 |  | 17**–**25 | 0.869 | 0.182 |  | 26**–**34 | 1 | 0 |  | 35**–**49 | 1 | 0 |
| *Nitrobacter* | 6**–**10 | 0.155 | 0.175 |  | 11**–**15 | 0.467 | 0.193 |  | 16**–**20 | 0.5 | 0.204 |  | 21**–**40 | 0.611 | 0.443 |
| *Nitrosococcus* | 4**–**8 | 0.0333 | 0.0745 |  | 9**–**14 | 0.12 | 0.138 |  | 15**–**19 | 0.762 | 0.146 |  | 20**–**27 | 0.777 | 0.403 |
| *Nitrosomonas* | 5**–**10 | 0.203 | 0.211 |  | 11**–**16 | 0.449 | 0.192 |  | 17**–**22 | 0.875 | 0.209 |  | 23**–**48 | 0.833 | 0.408 |
| *Nitrosospira* | 6**–**9 | 0.106 | 0.093 |  | 10**–**14 | 0.644 | 0.328 |  | 15**–**19 | 0.883 | 0.111 |  | 20**–**25 | 0.8 | 0.447 |
| *Nocardia* | 6**–**12 | 0.226 | 0.229 |  | 13**–**19 | 0.717 | 0.271 |  | 20**–**26 | 0.826 | 0.149 |  | 27**–**36 | 0.802 | 0.35 |
| *Nostoc* | 5**–**12 | 0.168 | 0.252 |  | 13**–**20 | 0.509 | 0.151 |  | 21**–**28 | 0.676 | 0.232 |  | 29**–**41 | 0.388 | 0.485 |
| *Oscillatoria* | 6**–**11 | 0.194 | 0.155 |  | 12**–**18 | 0.63 | 0.143 |  | 19**–**25 | 0.821 | 0.256 |  | 26**–**34 | 1 | 0 |
| *Pseudanabaena* | 6**–**12 | 0.161 | 0.203 |  | 13**–**19 | 0.778 | 0.246 |  | 20**–**26 | 0.952 | 0.125 |  | 27**–**41 | 0.875 | 0.353 |
| *Pseudomonas* | 7**–**12 | 0.255 | 0.389 |  | 13**–**18 | 0.691 | 0.196 |  | 19**–**24 | 0.885 | 0.18 |  | 25**–**35 | 0.773 | 0.368 |
| *Pseudonocardia* | 9**–**19 | 0.323 | 0.404 |  | 20**–**30 | 0.815 | 0.229 |  | 31**–**40 | 0.966 | 0.105 |  | 41**–**55 | 1 | 0 |
| *Rhizobium* | 5**–**9 | 0.125 | 0.19 |  | 10**–**15 | 0.222 | 0.178 |  | 16**–**21 | 0.376 | 0.287 |  | 22**–**28 | 0.805 | 0.305 |
| *Rhodobacter* | 7**–**14 | 0.216 | 0.357 |  | 15**–**22 | 0.848 | 0.217 |  | 23**–**30 | 0.921 | 0.175 |  | 32**–**48 | 1 | 0 |
| *Rickettsia* | 5**–**21 | 0.399 | 0.459 |  | 22**–**33 | 0.845 | 0.2 |  | 34**–**44 | 0.85 | 0.312 |  | 45**–**62 | 1 | 0 |
| *Shewanella* | 5**–**9 | 0.333 | 0.471 |  | 10**–**15 | 0.619 | 0.344 |  | 16**–**22 | 0.646 | 0.186 |  | 23**–**30 | 0.777 | 0.403 |
| *Sinorhizobium* | 4**–**10 | 0.16 | 0.158 |  | 11**–**15 | 0.288 | 0.208 |  | 16**–**20 | 0.616 | 0.273 |  | 21**–**30 | 0.7 | 0.447 |
| *Sphingomonas* | 6**–**13 | 0.135 | 0.274 |  | 14**–**21 | 0.689 | 0.327 |  | 22**–**29 | 0.907 | 0.202 |  | 30**–**40 | 1 | 0 |
| *Streptomyces* | 5**–**15 | 0.101 | 0.154 |  | 16**–**24 | 0.765 | 0.193 |  | 25**–**33 | 0.856 | 0.194 |  | 34**–**50 | 0.796 | 0.328 |
| *Variovorax* | 6**–**15 | 0.222 | 0.44 |  | 16**–**25 | 0.93 | 0.113 |  | 26**–**35 | 1 | 0 |  | 36**–**49 | 1 | 0 |
| *Xanthomonas* | 7**–**13 | 0.454 | 0.252 |  | 14**–**20 | 0.83 | 0.187 |  | 21**–**28 | 0.94 | 0.104 |  | 29**–**42 | 1 | 0 |
| AMF | 6**–**16 | 0.157 | 0.15 |  | 17**–**26 | 0.746 | 0.315 |  | 27**–**37 | 0.708 | 0.358 |  | 38**–**55 | 0.818 | 0.404 |
| *Aspergillus* | 6**–**10 | 0.405 | 0.234 |  | 11**–**15 | 0.24 | 0.205 |  | 16**–**20 | 0.116 | 0.162 |  | 21**–**37 | 0.2 | 0.447 |
| *Cenococcum* | 6**–**10 | 0.165 | 0.205 |  | 11**–**15 | 0.331 | 0.158 |  | 16**–**20 | 0.133 | 0.217 |  | 21**–**32 | 0.25 | 0.418 |
| *Cryptococcus* | 6**–**10 | 0.228 | 0.435 |  | 11**–**16 | 0.292 | 0.285 |  | 17**–**23 | 0.475 | 0.404 |  | 24**–**32 | 0.833 | 0.408 |
| *Mycosphaerella* | 4**–**8 | 0.266 | 0.326 |  | 9**–**13 | 0.56 | 0.308 |  | 14**–**18 | 0.518 | 0.153 |  | 19**–**26 | 0.542 | 0.366 |
| *Oidiodendron* | 5**–**9 | 0.15 | 0.223 |  | 10**–**14 | 0.325 | 0.129 |  | 15**–**19 | 0.526 | 0.345 |  | 20**–**25 | 0.533 | 0.505 |
| *Phanerochaete* | 6**–**9 | 0.471 | 0.11 |  | 10**–**14 | 0.424 | 0.157 |  | 15**–**19 | 0.373 | 0.127 |  | 21**–**30 | 0.6 | 0.547 |
| *Scleroderma* | 5**–**9 | 0.04 | 0.0894 |  | 10**–**14 | 0.287 | 0.149 |  | 15**–**19 | 0.388 | 0.146 |  | 20**–**36 | 0.9 | 0.223 |
| *Sebacina* | 5**–**9 | 0.0833 | 0.117 |  | 10**–**14 | 0.357 | 0.153 |  | 15**–**19 | 0.558 | 0.275 |  | 20**–**28 | 1 | 0 |

*Note*: Range represents a minimum and a maximum of (log_10_ (inverse of *P* value score)) values per quartile. After the result from each genus test set was divided into quartiles, the range of (log_10_ (Inverse *P* value score)) and the mean and the standard deviation of the correct prediction proportions were calculated per quartile. The result was based on the trimer usage probability scoring method. Data for Table S9 and Figure S5B.
