# Supplementary material for: SeSaMe: Metagenome Sequence Classification of Arbuscular Mycorrhizal Fungi-associated Microorganisms
Source: Genomics Proteomics Bioinformatics. 2020 Dec 18;18(5):601–12. doi: 10.1016/j.gpb.2018.07.010 (PMC8377386; doi:10.1016/j.gpb.2018.07.010)
Supplement: Supplementary Table S12 [file mmc12.doc]

**Table S12 Relationship between the correct prediction proportion of the rank probability scoring method and *P* value score in quartiles**

| **Genus** | **0–25^th^ percentiles** | | |  | **26–50^th^ percentiles** | | |  | **51–75^th^ percentiles** | | |  | **76–100^th^ percentiles** | | |
| --- | --- | --- | --- | --- | --- | --- | --- | --- | --- | --- | --- | --- | --- | --- | --- |
|  | **Range** | **Mean** | **SD** |  | **Range** | **Mean** | **SD** |  | **Range** | **Mean** | **SD** |  | **Range** | **Mean** | **SD** |
| *Acidithiobacillus* | 6–9 | 0.278 | 0.242 |  | 10–13 | 0.748 | 0.0505 |  | 14–17 | 0.968 | 0.0625 |  | 18–40 | 1 | 0 |
| *Acidobacterium* | 6–10 | 0.277 | 0.277 |  | 11–15 | 0.82 | 0.106 |  | 16–20 | 1 | 0 |  | 21–26 | 1 | 0 |
| *Agrobacterium* | 6–9 | 0.718 | 0.359 |  | 10–14 | 0.923 | 0.07 |  | 15–19 | 1 | 0 |  | 20–27 | 1 | 0 |
| *Anabaena* | 7–12 | 0.189 | 0.244 |  | 13–18 | 0.721 | 0.37 |  | 19–24 | 0.901 | 0.113 |  | 25–31 | 1 | 0 |
| *Azorhizobium* | 7–12 | 0.762 | 0.237 |  | 13–19 | 1 | 0 |  | 20–25 | 1 | 0 |  | 26–34 | 1 | 0 |
| *Azotobacter* | 6–11 | 0.567 | 0.343 |  | 12–17 | 0.894 | 0.117 |  | 18–24 | 1 | 0 |  | 25–43 | 0.833 | 0.408 |
| *Bacillus* | 5–11 | 0.244 | 0.308 |  | 12–18 | 0.77 | 0.226 |  | 19–24 | 0.777 | 0.194 |  | 25–37 | 1 | 0 |
| *Bdellovibrio* | 7–11 | 0.712 | 0.188 |  | 12–16 | 0.923 | 0.104 |  | 17–21 | 0.96 | 0.0894 |  | 22–27 | 1 | 0 |
| *Beijerinckia* | 5–10 | 0.6 | 0.383 |  | 11–15 | 0.9 | 0.173 |  | 16–20 | 1 | 0 |  | 21–34 | 1 | 0 |
| *Bradyrhizobium* | 7–11 | 0.712 | 0.209 |  | 12–16 | 0.95 | 0.0684 |  | 17–21 | 1 | 0 |  | 22–29 | 1 | 0 |
| *Caulobacter* | 7–11 | 0.689 | 0.317 |  | 12–16 | 0.971 | 0.0638 |  | 17–21 | 0.92 | 0.178 |  | 22–29 | 1 | 0 |
| *Clostridium* | 7–13 | 0.122 | 0.19 |  | 14–20 | 0.439 | 0.255 |  | 21–26 | 0.891 | 0.174 |  | 27–40 | 0.857 | 0.377 |
| *Cyanobacterium* | 11–17 | 0.516 | 0.273 |  | 18–24 | 0.823 | 0.262 |  | 25–31 | 0.976 | 0.0629 |  | 32-43 | 1 | 0 |
| *Desulfotomaculum* | 6–10 | 0.133 | 0.217 |  | 11–15 | 0.617 | 0.0981 |  | 16–20 | 0.893 | 0.153 |  | 22–35 | 1 | 0 |
| *Desulfovibrio* | 6–9 | 0.254 | 0.176 |  | 10–13 | 0.647 | 0.109 |  | 14–17 | 0.85 | 0.191 |  | 18–24 | 1 | 0 |
| *Erwinia* | 5–10 | 0.513 | 0.484 |  | 11–16 | 0.826 | 0.0847 |  | 17–22 | 1 | 0 |  | 23–30 | 1 | 0 |
| *Frankia* | 5–8 | 0.107 | 0.214 |  | 9–13 | 0.729 | 0.219 |  | 14–18 | 1 | 0 |  | 19–23 | 0.95 | 0.111 |
| *Geobacter* | 6–9 | 0.502 | 0.413 |  | 10–13 | 0.697 | 0.0762 |  | 14–17 | 1 | 0 |  | 18–22 | 0.9 | 0.223 |
| *Klebsiella* | 5–10 | 0.548 | 0.325 |  | 11–15 | 0.913 | 0.123 |  | 16–20 | 0.93 | 0.109 |  | 21–26 | 1 | 0 |
| *Kocuria* | 7–12 | 0.516 | 0.449 |  | 13–18 | 0.944 | 0.136 |  | 19–24 | 0.875 | 0.209 |  | 25–34 | 1 | 0 |
| *Leuconostoc* | 8–12 | 0.243 | 0.265 |  | 13–17 | 0.682 | 0.171 |  | 18–22 | 0.971 | 0.0638 |  | 23–27 | 1 | 0 |
| *Mesorhizobium* | 6–10 | 0.86 | 0.167 |  | 11–15 | 0.937 | 0.0908 |  | 16–20 | 1 | 0 |  | 21–28 | 1 | 0 |
| *Methylococcus* | 7–10 | 0.5 | 0.408 |  | 11–15 | 0.853 | 0.123 |  | 16–19 | 1 | 0 |  | 20–24 | 1 | 0 |
| *Microbacterium* | 7–11 | 0.4 | 0.418 |  | 12–17 | 0.877 | 0.113 |  | 18–23 | 1 | 0 |  | 24–32 | 1 | 0 |
| *Micrococcus* | 8–13 | 0.549 | 0.389 |  | 14–19 | 0.933 | 0.0831 |  | 20–24 | 1 | 0 |  | 25–35 | 1 | 0 |
| *Myxococcus* | 6–11 | 0.222 | 0.186 |  | 12–17 | 0.733 | 0.188 |  | 18–23 | 0.979 | 0.051 |  | 24–34 | 1 | 0 |
| *Nitrobacter* | 6–10 | 0.671 | 0.393 |  | 11–15 | 1 | 0 |  | 16–20 | 1 | 0 |  | 21–40 | 1 | 0 |
| *Nitrosococcus* | 5–8 | 0.583 | 0.3 |  | 9–13 | 0.752 | 0.11 |  | 14–17 | 0.937 | 0.125 |  | 18–24 | 1 | 0 |
| *Nitrosomonas* | 6–11 | 0.546 | 0.328 |  | 12–17 | 0.88 | 0.117 |  | 18–22 | 0.933 | 0.149 |  | 23–52 | 1 | 0 |
| *Nitrosospira* | 5–9 | 0.253 | 0.347 |  | 10–14 | 0.956 | 0.0603 |  | 15–19 | 1 | 0 |  | 20–26 | 1 | 0 |
| *Nocardia* | 6–10 | 0.283 | 0.298 |  | 11–16 | 0.767 | 0.183 |  | 17–21 | 0.98 | 0.0447 |  | 22–35 | 1 | 0 |
| *Nostoc* | 8–12 | 0.511 | 0.5 |  | 13–17 | 0.662 | 0.197 |  | 18–22 | 0.9 | 0.136 |  | 23–31 | 0.916 | 0.204 |
| *Oscillatoria* | 6–10 | 0.336 | 0.232 |  | 11–16 | 0.886 | 0.137 |  | 17–21 | 1 | 0 |  | 22–29 | 1 | 0 |
| *Pseudanabaena* | 6–12 | 0.236 | 0.29 |  | 13–19 | 0.927 | 0.0922 |  | 20–26 | 0.984 | 0.0419 |  | 27–38 | 1 | 0 |
| *Pseudomonas* | 6–9 | 0.602 | 0.225 |  | 10–14 | 0.807 | 0.155 |  | 15–19 | 0.981 | 0.0406 |  | 20–28 | 1 | 0 |
| *Pseudonocardia* | 7–13 | 0.553 | 0.375 |  | 14–19 | 1 | 0 |  | 20–24 | 1 | 0 |  | 25–32 | 1 | 0 |
| *Rhizobium* | 6–9 | 0.493 | 0.359 |  | 10–14 | 0.918 | 0.0784 |  | 15–18 | 0.958 | 0.0833 |  | 19–25 | 1 | 0 |
| *Rhodobacter* | 6–11 | 0.726 | 0.405 |  | 12–18 | 0.947 | 0.0899 |  | 19–24 | 1 | 0 |  | 25–38 | 1 | 0 |
| *Rickettsia* | 8–17 | 0.199 | 0.247 |  | 18–25 | 0.67 | 0.242 |  | 26–33 | 0.651 | 0.342 |  | 34–47 | 1 | 0 |
| *Shewanella* | 5–9 | 0.25 | 0.204 |  | 10–14 | 0.708 | 0.136 |  | 15–18 | 0.947 | 0.0611 |  | 19–27 | 1 | 0 |
| *Sinorhizobium* | 5–8 | 0.875 | 0.25 |  | 9–13 | 0.923 | 0.104 |  | 14–18 | 0.98 | 0.0447 |  | 19–24 | 1 | 0 |
| *Sphingomonas* | 6–11 | 0.56 | 0.347 |  | 12–17 | 0.883 | 0.204 |  | 18–24 | 0.972 | 0.068 |  | 25–33 | 1 | 0 |
| *Streptomyces* | 5–10 | 0.139 | 0.219 |  | 11–16 | 0.863 | 0.141 |  | 17–22 | 1 | 0 |  | 23–32 | 1 | 0 |
| *Variovorax* | 7–12 | 0.291 | 0.367 |  | 13–18 | 0.715 | 0.172 |  | 19–24 | 0.986 | 0.034 |  | 25–31 | 1 | 0 |
| *Xanthomonas* | 7–11 | 0.516 | 0.207 |  | 12–17 | 0.839 | 0.202 |  | 18–23 | 1 | 0 |  | 25–39 | 1 | 0 |
| AMF | 6–11 | 0.0654 | 0.106 |  | 12–18 | 0.502 | 0.284 |  | 19–25 | 0.678 | 0.386 |  | 26–35 | 0.785 | 0.393 |
| *Aspergillus* | 5–8 | 0.398 | 0.377 |  | 9–12 | 0.87 | 0.0933 |  | 13–16 | 0.873 | 0.148 |  | 17–26 | 0.75 | 0.5 |
| *Cenococcum* | 6–9 | 0.625 | 0.25 |  | 10–13 | 0.82 | 0.208 |  | 14–17 | 0.703 | 0.216 |  | 18–41 | 0.72 | 0.414 |
| *Cryptococcus* | 6–9 | 0.339 | 0.282 |  | 10–14 | 0.61 | 0.286 |  | 15–18 | 0.825 | 0.236 |  | 19–25 | 0.8 | 0.447 |
| *Mycosphaerella* | 6–9 | 0.816 | 0.137 |  | 10–13 | 0.83 | 0.05 |  | 14–17 | 0.895 | 0.125 |  | 18–25 | 1 | 0 |
| *Oidiodendron* | 6–8 | 0.555 | 0.509 |  | 9–12 | 0.794 | 0.151 |  | 13–16 | 0.958 | 0.0833 |  | 17–22 | 0.816 | 0.213 |
| *Phanerochaete* | 5–7 | 0.154 | 0.135 |  | 8–11 | 0.488 | 0.213 |  | 12–15 | 0.78 | 0.182 |  | 16–21 | 0.775 | 0.262 |
| *Scleroderma* | 6–10 | 0.474 | 0.245 |  | 11–15 | 0.679 | 0.164 |  | 16–20 | 0.971 | 0.0638 |  | 21–30 | 1 | 0 |
| *Sebacina* | 7–9 | 0.466 | 0.416 |  | 10–13 | 0.69 | 0.183 |  | 14–17 | 0.843 | 0.119 |  | 18–23 | 1 | 0 |

*Note*: Range represents a minimum and a maximum of (log_10_ (inverse of *P* value score)) values per quartile. After the result from each genus test set was divided into quartiles, the range of (log_10_ (inverse *P* value score)) and the mean and the standard deviation of the proportions of the correct taxon group were calculated per quartile. The result was based on the rank probability scoring method. Data for Table S10 and Figure S5C.
